# A scoping review of computational models of the diabetic foot

**DOI:** 10.1371/journal.pone.0351638

**Published:** 2026-06-15

**Authors:** Yufeng Li, Athia Haron, Chaofan Lin, Yuan Tang, Andrew Weightman, Glen Cooper

**Affiliations:** Department of Mechanical and Aerospace Engineering, University of Manchester, Manchester, Greater Manchester, United Kingdom; Chongqing University Three Gorges Hospital, CHINA

## Abstract

The prevalence of diabetes is expected to be 650 million people by 2030, and diabetic foot ulceration (DFU) is one of its most severe complications. It poses a significant challenge to global health and brings substantial social and economic burdens. Although many studies have explored the mechanisms of DFU development, they are still not fully understood. Due to the high cost of the experimental research, many recent studies have employed the computational modelling approaches to simulate the effects of diabetes on foot tissues from mechanical, thermal, fluid, and cellular perspectives. This study aims to provide a comprehensive review of computational modelling approaches used to investigate various factors influencing DFU, discuss current knowledge gaps and limitations, and outline future research directions. A systematic search was conducted in Web of Science, Scopus, and PubMed databases, identifying a total of N = 1631 records up to March 2025, 31 of which studies met the inclusion criteria and were analysed in this study. Results showed that DFU-related computational models can be categorized into five types: mechanical stress models, thermal models, vascular and nerve system models, multiphysics models, and cellular-based models. These models explore the formation mechanisms of DFU from different perspectives, including biomechanics, temperature, fluid dynamics, HHμm neural signalling, and cellular responses. However, except for mechanical stress models, the other approaches remain in the early stages of development, and the single physics modelling strategies are unable to provide understanding on the coupled processes with the foot and their effect on DFU. Future research should further develop modelling approaches and couple these together to develop comprehensive understanding of DFU pathogenesis.

## Introduction

### Background

In recent years, diabetes has become an important worldwide problem that cannot be ignored [1]. According to statistics from the International Diabetes Federation (IDF), in 2024, there were 587 million people with diabetes worldwide, representing 11% of the global population [2]. Additionally, the number of people suffering from diabetes and its prevalence rate is still rising, and it is expected that by 2050, 853 million people worldwide will suffer from diabetes, with a prevalence rate of more than 12% [2]. At the same time, diabetes places a huge burden on the national economy, with the National Health Service (NHS) in the UK spending £19,000 per minute on diabetes [3].

Diabetes leads to elevated blood sugar and metabolic disorders [4], which can lead to multiple organ complications and, in severe cases, kidney failure, stroke, disability and even death [5].

Among all the complications of diabetes, diabetic foot ulceration (DFU) is the most costly and destructive [6]. DFU is a chronic, non-healing ulcer of the lower limbs that occurs in patients with diabetes mellitus (DM), primarily as a result of long-standing disease, poor glycaemic control, and damage to the peripheral blood vessels and nerves [6,7]. DFU can lead to hard-to-heal wounds on the plantar foot, which can greatly increase the patient’s risk of infection and amputation [4].

Around 15% of diabetes mellitus will have DFU during their lives [6]. According to the statistics, the mortality rate of normal DFU patients within 5 years is 30%, and for the amputated DFU patients, the rate can be up to 70% [8], which makes DFU second only to cancer in terms of mortality [9], and as far back as 2017, the NHS’s estimated spending on diabetic group ulcers have already reached £935 million annually [10].

This shows that it is particularly important to find the pathological mechanisms of DFU and to develop an effective treatment plan for the patients.

### Key investigated factors in DFU and modelling methods

It is now widely recognised that plantar neuropathy, blockage of the foot arteries and infection of hard-to-heal wounds caused by high blood sugar are the three main pathophysiologies of DFU [11–13]. However, the potential triggers of DFU and the mechanisms of its development are not fully understood [14].

It has been demonstrated that, due to increased body weight and altered material properties of the plantar soft tissues [15], diabetes mellitus has increased plantar pressure distribution [14,16,17], greater peak pressure [18] and shear forces [14] within the tissue. This increases the possibility of DFU formation, which is why many studies [19–21] have been devoted to the development of pressure-reducing insoles to improve the treatment of diabetic foot. Additionally, temperature has also been shown to be an important factor in the formation of DFU [22,23], with studies showing that the plantar temperature of people living with diabetes is 1.2°C higher than healthy individuals, and a temperature difference of more than 2.2°C between the same locations on both feet can be an early indicator of ulceration development [24]. Higher plantar temperatures are more likely to lead to deep tissue damage under the same conditions of pressure [25]. In recent years, some studies [26–28] also have begun to focus on the impact of changes in plantar cell behaviour on DFU, and it has now been demonstrated that changes in skin composition [27] and adipocyte morphology [29] can have an impact on the formation of DFU.

Computational models have also been used to study DFU. Among them, constitutive or numerical models [30,31] are often used to define the hyperelastic and viscoelastic properties of plantar soft tissues, finite element analysis (FEA) [32–34] is widely used for pressure distribution or temperature analysis of the plantar foot because of the flexibility of parameter settings and the characteristics of multi-scale analysis, and computational fluid dynamics (CFD) [35] is used to analyse the effects of vascular lesions and the blood flow on DFU. A few studies have also used agent-based models [26] to simulate the microscopic behaviour of plantar cells and media, in order to gain a comprehensive understanding of the effects of DFU on plantar tissues, studies have also been conducted to apply multiphase models [35,36].

Although the computational models have contributed to the understanding of the development and progression of DFU from different perspectives, existing studies still show substantial differences and limitations in terms of the factors considered, modelling scales, and underlying assumptions. On the one hand, many models focus on only a single DFU influencing factor, such as mechanical stress, temperature changes, or blood flow, which makes it difficult to fully represent the complex and multifactorial nature of DFU [26,32–35]. On the other hand, the spatial scales of these models vary widely, which range from the macroscopic structure of the foot to microscopic tissue and even cellular levels, and clear links between models at different scales are still lacking [26,32–35]. Therefore, to systematically review existing studies and clarify their applicability and limitations, this review organizes DFU-related computational models along two complementary dimensions. The first dimension is based on the main biomechanical or biological processes emphasized by the model, which reflects the dominant factors considered in DFU research. The second dimension is based on the spatial scale of the model, distinguishing different levels of representation from the whole foot to local tissue regions and cellular scales. This classification helps highlight differences in research focus and modelling assumptions among existing studies and provides a structured framework for the comparative analysis.

### Aim and objectives

This review aims to systematically assess computer models in the literature to understand the different factors that have been considered related to DFU. The following research question was formulated: What types of computational models have been developed for DFU, what DFU-related mechanisms do they capture, and how do these models contribute to clinical understanding and potential solutions, while highlighting the current state and limitations of the field?

The objectivities of this review are to:

1. Summarise the models that focus on different DFU factors: such as mechanical modelling, thermal modelling, blood flow modelling, nerve system modelling, agent-based cellular modelling, and multiphysics modelling.
2. Determine the knowledge gaps and limitations of current research on foot modelling.

## Methods

A scientific literature search was conducted in three databases Web of Science, Scopus and PubMed around computational modelling of foot or plantar tissues. The following search terms were used in three databases, due to differences in indexing systems across databases, the specific search terms vary slightly between the three databases; however, all search strategies were developed based on a consistent underlying logic and conceptual framework to ensure comparability and comprehensiveness of the search:

1 **Web of Science:** ((TS=(plantar) OR TS=(plantar foot) OR TS=(foot sole) OR TS=(plantar soft tissue*) OR TS=(plantar skin) OR TS=(foot skin) OR TS=(diabetic foot) OR TS=(diabetic feet) OR TS=(neuropathic foot) OR TS=(diabetic foot ulcer*) OR TS=(foot ulcer*) OR TS=(diabetic soft tissue*) OR TS=(plantar tissue mechanic*) OR TS=(foot tissue mechanic*))

AND

((TS=(finite element*) OR TS=(finite element model*) OR TS=(FE model*) OR TS=(FE analysis*) OR TS=(FEA) OR TS=(FEM) OR TS=(Mechanobiological model*) OR TS=(constitutive model*) OR TS=(computational model*) OR TS=(computational model*) OR TS=(fluid dynamic*) OR TS=(CFD) OR TS=(fluid model*) OR TS=(two phase model*) OR TS=(multi-phase model*) OR TS=(thermodynamic model*) OR TS=(thermal model*) OR TS=(temperature model*) OR TS=(multiphysics model*) OR TS=(computational fluid dynamics)) OR ((TS=(diabetic foot) OR TS=(skin wound)) AND TS=(agent based model*)))

NOT

(TS = (insole*) OR TS = (shoe*) OR TS = (footwear*) OR TS = (device*) OR TS = (sensor*) OR TS = (deformity) OR TS = (hammer toe*) OR TS = (flatfoot) OR TS = (ostrich foot) OR TS = (amputation) OR TS = (machine learning) OR TS = (deep learning)).

2 **Scopus:** ((TITLE-ABS-KEY(“plantar” OR “plantar foot” OR “foot sole” OR “plantar soft tissue*” OR “plantar skin” OR “foot skin” OR “diabetic foot” OR “diabetic feet” OR “neuropathic foot” OR “diabetic foot ulcer*” OR “foot ulcer*” OR “diabetic soft tissue*” OR “plantar tissue mechanic*” OR “foot tissue mechanic*))

AND

(TITLE-ABS-KEY(“finite element*” OR “finite element model*” OR “FE model*” OR “FE analysis*” OR “FEA” OR “FEM” OR “mechanobiological model*” OR “constitutive model*” OR “computational model*” OR “fluid dynamic*” OR “CFD” OR “fluid model*” OR “two phase model*” OR “multi phase model*” OR “thermodynamic model*” OR “thermal model*” OR “multiphysics model*” OR “blood flow model*” OR “computational fluid dynamics”))) OR (TITLE-ABS-KEY (“diabetic foot” OR “skin wound*”) AND (TITLE-ABS-KEY (“agent based model*”)))

AND NOT

(TITLE-ABS-KEY (insole*) OR TITLE-ABS-KEY (shoe*) OR TITLE-ABS-KEY (footwear*) OR TITLE-ABS-KEY (device*) OR TITLE-ABS-KEY (sensor*) OR TITLE-ABS-KEY (deformity) OR TITLE-ABS-KEY (hammer AND toe*) OR TITLE-ABS-KEY (flatfoot) OR TITLE-ABS-KEY (ostrich AND foot) OR TITLE-ABS-KEY (amputation) OR TITLE-ABS-KEY (deep learning) OR TITLE-ABS-KEY (machine learning))

3 **PubMed:** ((“foot”[MeSH Terms] OR “foot”[All Fields]) AND (“diabetes mellitus”[MeSH Terms] OR (“diabetes”[All Fields] AND “mellitus”[All Fields]) OR “diabetes mellitus”[All Fields] OR “diabetes”[All Fields] OR “diabetic”[All Fields])

AND

((“finite”[All Fields] AND (“elements”[MeSH Terms] OR “elements”[All Fields] OR “element”[All Fields])) OR ((model[All Fields] OR modeling[All Fields]) AND (“mechanobiological”[All Fields] OR “constitutive”[All Fields] OR “fluid”[All Fields] OR “computational”[All Fields] OR “two phases” OR “three phases”[All Fields] OR “thermodynamic”[All Fields] OR “thermal”[All Fields] OR “multiphysics” OR “blood flow”[All Fields] OR “temperature”[All Fields])))) OR ((“diabetic foot”[All Fields] OR “skin wound”[All Fields]) AND (“agent based”[All Fields]))

NOT

(“insole”[All Fields] OR “shoe”[All Fields] OR “footwear”[All Fields] OR “device”[All Fields] OR “sensor”[All Fields] OR “deformity”[All Fields] OR “hammer toe”[All Fields] OR “flatfoot”[All Fields] OR “ostrich foot”[All Fields] OR “amputation”[All Fields] OR “deep learning”[All Fields] OR “machine learning”[All Fields]).

The literature search included all relevant studies published up to March 2025. The search was restricted to full-text, English-language, and human studies. Additionally, the reference lists of the included articles were screened to identify any additional eligible studies. After removing duplicates and records without accessible full text, titles and abstracts were screened according to the inclusion and exclusion criteria, and the full texts of potentially eligible articles were assessed.

The initial search results contained many articles primarily focused on medicine or pharmacy, so the following inclusion and exclusion criteria were imposed on the search results by reviewing the titles, abstracts, and main texts of the articles respectively.

The inclusion criteria for this study were:

1. Articles related to the analysis of foot tissue or diabetic foot or foot ulcers using finite element methods or other computational modelling methods (agent-based model, numerical model, multiphysics model, thermal model, fluid model).
2. Determined the material properties of the plantar soft tissues.
3. Investigated the stress and strain distribution, temperature, blood flow, nerve system or the cellular behaviour of the plantar soft tissues.

The exclusion criteria for this study were:

1. Articles not related to foot modelling.
2. Articles primarily focused on clinical medicine, pharmacology, veterinary science, chemistry, nursing, environmental science, social sciences, decision sciences, or arts and humanities were excluded when they did not involve computational modelling, biomechanical analysis, or engineering-based approaches relevant to DFU.
3. Articles that did not focus on the plantar tissues, such as only the Achilles tendons, ligaments, ankles or bones.

After the study selection process was completed, the included articles were analysed using a structured grouping approach. Specifically, studies were categorised according to the DFU-related physiological or biomechanical parameters they addressed, as well as the type of computational model employed. Within each group, a comparative analysis was conducted focusing on modelling strategies, material properties, boundary condition settings, as well as the strengths, limitations, and current state of development of the models.

## Results

A total of 1631 articles were identified from the three databases (Web of Science: 1173, Scopus: 245, PubMed: 213), and after screening out 324 duplicates and 59 results that were not accessible online, the titles and abstracts of the remaining 1248 articles were reviewed to exclude 1187 articles according to the inclusion and exclusion criteria.

**Fig 1:**
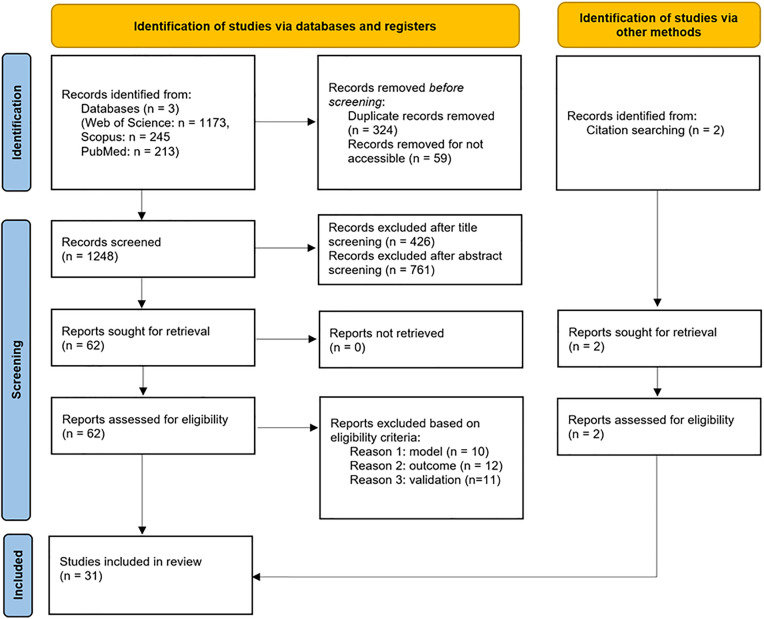
Study identification flow chart. Template was taken from PRISMA..

A total of 62 articles were retrieved for full-text review. These articles were independently assessed by two reviewers (Li and Tang). In cases where disagreements arose regarding study inclusion, the disputed articles were further evaluated by a third reviewer (Lin) to reach a consensus. Following this literature selection and review process, 33 were excluded for the following reasons: (a) the model was not developed using appropriate geometry medical imaging or experimental data; (b) the study outcomes were not relevant to advancing the understanding of diabetic foot ulcers; and (c) the model lacked adequate validation. In addition, two relevant articles were identified through screening the reference lists of the included studies. Ultimately, a total of 31 articles were included in the final analysis. The overall selection process and results are presented in Fig 1.

Since the selected articles show diversity in foot modelling, this paper carried out two classifications of the foot models in the selected articles. The first classification is based on the levels of modelling from macroscopic to microscopic. Specifically, there are two-dimensional (2D) cross-sectional models, three-dimensional (3D) whole foot models [32–43], specific region models [30,44–49], tissue level models [31,50–55] and micro-level models [27,56–59].

Both the 2D cross-sectional models [32] and the 3D whole foot models [33–43] can represent the complete foot structure including the Achilles tendon, the difference being only that the former is a two-dimensional anatomical model along the metatarsal rays, and the latter represents the spatial geometry of the whole foot. On the other hand, specific region models [30,44–49] are the models that represent the behaviour of specific regions of the foot (for example, the forefoot or the heel).

In contrast to these models, tissue-level models [31,50–55] focus on the unique structure of the plantar soft tissues (adipose tissue, muscle tissue, skin), and cellular models [27,56–59] discuss behaviour and changes at the cellular level of the plantar foot.

Additionally, this study categorised the models into mechanical stress models [30–34,38,39,44–47,49–52,55], thermal models [40–42], vascular and nerve models [48,53,54], multiphysics models [35–37,43] and agent-based models [27,56–59] according to the research focus that the models are concerned with. The included studies are summarized by these groups in Table 1.

**Table 1:** Studies on the different levels of foot computer modelling.

Source	Study Context	Primary Research Theme/Objective	Model			Experimental Setting	Feature(s)
			Classification	Component(s)	Validation status		
Actis RL et al (2006)	Experimental–computational (USA)	Develop second- and third-foot rays model and investigate the model parameters sensitivity	M, C1	Bone, cartilage, flexor tendon, fascia, soft tissues	Experiment validated (stress measurement insole)	**Participants:** n = 6, 5 subjects with diabetes and plantar ulcer history, 1 subject in healthy condition. **Equipment:** spiral X-ray computed tomography for foot structure, F-scan system for pressure distribution. **Boundary conditions:** 50% body weight	**Key Findings:** Pressure under the second metatarsal is larger, and footwear has a significant effect on stress reduction. **Limitations:** Ignored the spatial impact of foot soft tissues and dynamic shear surface forces
Chen et al (2010)	Experimental-computational (Australia)	Develop a 3D foot model and investigate plantar soft tissue deformation and stress	M, C2	Bone, cartilage, ligament, plantar fascia, plantar soft tissue	Experiment validated (stress measurement insole)	**Participants:** n = 1, a male subject, healthy condition, age 26. **Equipment:** F-scan for plantar pressure. MIMICS and PATRAN for finite element. **Boundary conditions:** stand barefoot on a metal plate, 100% body weight.	**Key Findings:** The contact region of soft tissues and MTH has large von Mises stress, and the bony prominences have a significant effect on internal stress distribution. **Limitations:** Ignored the changes in material properties.
Fontanella et al (2017)	Experimental-computational (Italy)	Investigate the mechanical behaviour of the healthy and degenerative plantar soft tissues	M, C2	Bones, cartilage, muscles, connective tissues, skin	Experiment validated (force plate data)	**Participants:** n = 1, a female subject, healthy condition, age 23 (from previous study) **Equipment:** AMTI BP400600 force plates for ground reaction forces, Vicon MX system for gait recording. **Boundary conditions:** standing in barefoot and the shod condition, walking at 1.6m/s in barefoot and shod condition.	**Key Findings:** Tissue degeneration increases stiffness and reduces energy absorption ability; spreads pressure more evenly. **Limitations:** Simplified model geometry.
Mo et al (2019)	Experimental-computational (China)	Investigate the stiffness and thickness variation of soft tissues, the effect of ageing and gender	M, C2	Bones, Achilles tendon, ankle ligaments, fascia, micro/macro chamber	Experiment validated (vertical compression tests)	**Participants:** n = 50, 25 female subject, 25 male subjects, healthy condition, age 20–69. **Equipment:** Aixplorers ultrasound system for soft tissue elasticities of different stiffness, MAGNETOM Prisma 3.0T for MRI images. **Boundary conditions:** standing in barefoot conditions (700N vertical ground reaction force)	**Key Findings:** With increased age, the stiffness of micro/macro chambers and the whole heel pad slightly decreased, and both stiffness and thickness of male plantar tissues were slightly larger than females. **Limitations:** Only based on a single patient’s MRI scan
Bocanegra et al (2020)	Experimental-computational (Spain)	Investigate the plantar pressure changes in the second rocker gait	M, C2	bones, skin, cartilage, ligament, fascia, soft tissues, muscle	Compared with literature	**Participants:** n = 1, health condition, age 40. **Equipment:** CT and MRI images for foot modelling. **Boundary conditions:** standing in barefoot conditions	**Key Findings:** Peak plantar pressure is located at the heel centre and a model that considers the sliding mechanism can show more accurate stress distribution. **Limitations:** Time consuming.
Fontanella et al (2014)	Experimental-computational (Italy)	Investigate the mechanical behaviour of the forefoot soft tissues	M, C3	Bones, soft tissue, skin, cartilag0.. e	Experiments validated (indentation tests)	**Participants:** n = 20, healthy condition, age 55.91 ± 10.97 **Equipment:** indentation experimental test system with a cylindrical intender (diameter = 7.9 mm). **Boundary conditions:** constant displacement rate of 18 mm/s.	**Key Findings:** Determined soft tissue constitutive formulation. **Limitations:** Lack of experiments.
M. Kardeh et al. (2016)	Experimental-computational (German)	Characterize the mechanical behaviour of plantar soft tissues under impact	M, C3	Calcaneus, muscle, Achilles tendon, fat pad	Experiment validated (heel pad deformation)	**Participants:** n = 1, a female subject, healthy condition, age 25. **Equipment:** MAGNETOM SONATA MRI system; custom loading device; force transducer; ABAQUS finite element software. **Boundary conditions:** Foot fixed (28°), quasi-static: displacement → 9 mm with 90 s hold; friction 0.4; calcaneus fixed. Dynamic impact: mass 980 g; velocity 1040–1480 mm/s; repeated 5 times.	**Key Findings:** successfully characterized the human heel pad’s mechanical behaviour by using a combined in vivo and numerical approach. **Limitations:** based on a subject-specific model, didn’t consider the bone movement.
Ahanchian et al (2017)	Experimental-computational (UK)	Investigate the material properties of the heel pad sub-layers	M, C3	Muscle tissue, plantar fascia, heel pad sublayers	Compared loaded heel thickness with MRI images	**Participants:** n = 1, a female subject, healthy condition, age 34. **Equipment:** Philips 1.5T Acheiva for MRI images, STRIDE (an indentation test platform) for investigating the strain. **Boundary conditions:** slow compression (5 mm/s), rapid compression (225 mm/s).	**Key Findings:** Determined optimized hyperelastic & viscoelastic soft tissue material properties. **Limitations:** Same time constraints for all soft tissues’ layers, Ignored shear loadings.
Friedman et al (2019)	Experimental-computational (Israel)	Investigate the foot injury load threshold	M, C3	Skin, soft tissue, Achilles tendon, calcaneus, cartilage	Experiment validated (in vivo experiments)	**Participants:** n = 1, a male subject, healthy condition, age 72. **Equipment:** MRI scan images. **Boundary conditions:** foot resting on the support at Trendelenburg position (5, 10, 20, 30 degrees), horizontal position, reverse Trendelenburg position (5, 10, 29, 30 degrees).	**Key Findings:** Injury threshold of fat layer is much lower than skin, shear force play more than 50% role in plantar foot injury. **Limitations:** Only a subject-specific study.
Trebbi et al (2023)	Experimental-computational (France)	Develop a model for diabetic foot ulceration prevention	M, C3	Bones, tendons, adipose tissues, muscle, skin	Experiment validated (indentation test)	**Participants:** n = 1, a male subject, healthy condition, age 40. **Equipment:** an indenting platform. **Boundary conditions:** compression test (0N, 15N, 140N), shear test (15N normal and 45N shear, 15N normal and 12N shear)	**Key Findings:** Fat pad becomes stiffer with increased loadings, fat region close to bone prominence has higher strain. **Limitations:** Subject-specific study, Ignored viscous behaviour of soft tissues.
Zhang et al (2023)	Experimental-computational (China)	Develop a model to predict Internal heel pad tissue stresses	M, C3	Skin, soft tissues, intrinsic muscles and calcaneus	Experiment validated (gait experiment)	**Participants:** n = 1, a female subject, diabetic condition, age 66. **Equipment:** the integrated DFIS-pressure platform. **Boundary conditions:** walking at 1.0m/s.	**Key Findings:** Peak plantar pressure is located at fat – bone interface of the heel centre, tensile stresses found on the skin interfaces, diabetic ulceration may start in deeper subcutaneous tissue layers. **Limitations:** Ignored whole foot structure
Qian et al (2010)	Experimental-computational (China)	Investigate the heel pad Impact attenuation and absorption mechanism	M, C4	fat cells, reticular fibre structure)	Experiment validated (gait experiment)	**Participants:** n = 1, a male subject, healthy condition, age 27. **Equipment:** Qualiys (a 12 infrared camera motion analysis system) for capturing the 3D motions at 150 Hz, Kistler (a 6-force plate array) for measuring the ground reaction forces. **Boundary conditions:** walking at normal speed in barefoot along an indoor walkway.	**Key Findings:** The coupling mechanism can reduce plantar stress centralization. **Limitations:** The model is simplified and subject specific.
Natali et al (2012)	Experimental-computational (Italy)	Determine the heel pad deformation and mechanical properties’ relationship	M, C4	Adipose chambers and connective septae)	Experiment validated (pig tissue compression experiment)	**Participants:** no human participants, model geometry parameter found from other literture. **Equipment:** not mentioned in the paper. **Boundary conditions:** unconfined compression test (quasi-static: 0.01 mm/s and 0.001 mm/s to 50% strain, rapid: 175 mm/s and 350 mm/s to 50% strain)	**Key Findings:** Bigger adipose chambers can cause higher stresses on connective septae. **Limitations:** Ignored the effect of ageing or pathologies.
Fontanella et al (2016)	Experimental-computational (Italy)	Investigate the relationship of morphological configuration and fat pad behaviour	M, C4	Adipose chambers and connective septae	Experiment validated (indentation test)	**Participants:** no human participants, model geometry parameter found from other literature, and dead human tissue. **Equipment:** not mentioned in the paper. **Boundary conditions:** unconfined compression test (constant speed: 40 mm/s)	**Key Findings:** Degraded tissues have larger stiffness modulus and peak stress, with developed compressive stress in fat tissue and tensile stress in skin tissue. **Limitations:** Homogenous model
Ou et al (2018)	Experimental-computational (China)	Develop the constitutive model of the plantar soft tissue.	M, C4	Skin, soft tissues (adipose and connective septae), bones	Experiment validated (indentation test)	**Participants:** dead body tissue (n = 2, age 50–60, weight:60–70 kg). **Equipment:** optical microscopy (Zeiss, LSM 700) for tissue details **Boundary conditions:** unconfined compression test (to 30% strain)	**Key Findings:** Under compressive load, adipose tissue exhibits bulging and vertical deformation, while connective septae stiffen plantar soft tissues, leading to increased compressive stiffness. **Limitations:** Cannot represent fibre connectivity accurately.
Kwak et al (2020)	Experimental-computational (Korea)	Evaluate the mechanical behaviours of plantar soft tissues	M, C4	Skin and fat	Experiment validated (indentation test)	**Participants:** n = 30, 10 healthy young participants (age 25 ± 1.4), 10 healthy elder participants (age 65.4 ± 7.5), 10 diabetic elder participants (age 65.0 ± 7.5). **Equipment:** custom foot sole indentation device, Reality Capture system for foot model construction **Boundary conditions:** indentation test (1 mm/s till the depth to 8 mm)	**Key Findings:** Elder people with diabetes have softer skin, stiffer fat. **Limitations:** Fat layer as one bulk material and didn’t consider viscoelasticity of plantar tissues.
Boyle et al (2019)	Experimental-computational (UK)	Investigate the morphology and composition of plantar skin under the loadings	M, C5	Skin (stratum corneum, viable epidermis, and dermis)	Experiment validated (microscope observation and compression tests)	**Participants:** n = 12 (2 donors × 2 sites × 3 samples per site). **Equipment:** Biomomentum Mach-1 v500 cst; ATI Nano 17 F/T18874 force transducer **Boundary conditions:** Compression (0.005 s ^−1^ → 10 kPa) + shear (0.005 s ^−1^ → 2 kPa), with preconditioning cycles and 300 s creep hold	**Key Findings:** Plantar stratum corneum reduces tissue pressure, epidermis and dermis reduce the deformation, decreased collagen and elastin synthesis can lead to pressure ulceration. **Limitation:** focus on the plantar skin only
Durany et al (2013)	Computational (Spain)	Investigate the heat transfer of the human foot	T, C2	Skin and other components, socks	Not validated	**Participants:** no participants **Equipment:** no equipment, COMSOL for the simulation. **Boundary conditions:** perfect contact between foot and textile medium	**Key Findings:** Determined a thermodynamics equation of foot heat transfer. **Limitations:** used a highly simplified model.
Bayareh et al (2016)	Computational (Mexico)	Investigate the heat distribution of skin on deep plantar foot	T, C2	Whole foot model (skin, muscles and other components)	Not validated	**Participants:** model was constructed based on 160 male subjects with ages between 18 and 25-year-old. **Equipment:** no equipment, COMSOL for the simulation. **Boundary conditions:** the bioheat and the heat transfer in fluids for the surrounding air was simulated	**Key Findings:** Higher temperature at ulcers region, ulcers can quickly heat the surrounding area, heat cannot dissipate over time. **Limitations:** Ignored the multi-layer structure of the foot and no validation experiments were done, all ulcer areas were placed at the same distance from the sole.
Copetti et al (2017)	Computational (Brazil)	Investigate the foot blood flow, the effect of sweating	T, C2	Whole foot	Compared with literature	**Participants:** no participants **Equipment:** no equipment, COMSOL for the simulation. **Boundary conditions:** bioheat transfer	**Key Findings:** Foot temperature is lowest at toes and is more pronounced with lower temperature. **Limitations:** ignored effect of diabetes.
Hahn et al (2007)	Computational (USA)	Investigate the effect of tissue stiffness, damping and external force on arteries	F, C3	The big toe of the foot	Experiments validated and compared with literature	**Participants:** no participants **Equipment:** no equipment. **Boundary conditions:** numerical simulation, not mentioned	**Key Findings:** Hardening of the plantar tissue in diabetic patients leads to constriction of the arteries and reduced blood flow. **Limitations:** ignored the exact structure of the foot
Wang et al (2021)	Computational (UK)	Investigate the effect of plagues on behaviours of blood	F, C4	Blood vessels and blood flow	Compared with literature	**Participants:** n = 1, a male subject, stenosed lower limb vessels, age 73 **Equipment:** no equipment, MIMICS for modelling. **Boundary conditions:** One-way FSI; fixed ends; bound wall–plaque contact; outer pressure 1 kPa; pulsatile inlet velocity & outlet pressure (0.8 s cycle)	**Key Findings:** The initiation of new plaques may occur in healthy areas behind lipid plaques and tend to accumulate further around calcified plaques. **Limitations:** All materials built as isotropic.
Haque et al (2022)	Computational (Pakistan)	Develop a functional dorsal nerve model	F, C4	Foot nerve system	Not validated	**Participants:** no participants **Equipment:** no equipment. **Boundary conditions:** numerical simulation, not mentioned	**Key Findings:** Developed the first computational combined functional dorsal nerve model. **Limitations:** no validation experiments.
Fernandez et al (2012)	Experimental-computational (New Zealand)	Present an efficient computational model of the foot	P, C2	Bones, soft tissues, muscles, skin, nerve system	Experiment validated (human motion capture), compared with literature	**Participants:** details not mentioned. **Equipment:** Optitrack motion capture system (12 cameras, 100 fps); Gaitway instrumented treadmill with Kistler force plates; emed pressure platform. **Boundary conditions:** Walking at ~1.2 m/s; three gait phases (heel strike, midstance, toe-off); synchronized kinematics, force, and plantar pressure data.	**Key Findings:** A slight increase in peak plantar pressure is associated with pathological sclerosis of the soft tissue; tissue damage begins in the deeper tissues rather than the skin. **Limitations:** Assumed that healthy people and DFU patients have similar gait, all muscle fibres are aligned in the longitudinal direction.
Mithraratne et al (2012)	Experimental-computational (New Zealand)	Investigate the pathologic foot’s blood flow	P, C2	Bones, soft tissues, blood vessels	Compared with literature	**Participants:** one male subject, details not mentioned. **Equipment:** 12-camera motion capture system, force plate **Boundary conditions:** Walking (gait cycle, details not mentioned)	**Key Findings:** Pathologic foot causes a reduction in blood vessel radius and an increase in hydrostatic pressure. **Limitations:** Ignored microvasculature and venous network for the return flow.
Sciumè et al (2014)	Computational (Italy)	Develop a model for diabetic foot ulceration prevention	P, C2	Bone, soft tissues (matrix, cells)	Compared with literature	**Participants:** details not mentioned. **Equipment:** not mentioned **Boundary conditions:** no experiments.	**Key Findings:** Peak plantar pressure is located at the heel centre. **Limitations:** The model did not consider the multi-layer structure of the plantar soft tissues.
Wang et al (2022)	Experimental-computational (China)	Investigate the effect of diabetes on blood vessels and blood flow	P, C2	Bones, soft tissues. Blood vessels	Experiment validated (human participants and animal experiments)	**Participants:** details not mentioned. **Equipment:** developed portable thermal sensor. **Boundary conditions:** Subject stationary; 3 phases—rest (0 W/m², 750 s), heating (150 W/m², 1000 s), recovery (0 W/m², 1000 s).	**Key Findings:** Diabetes causes delayed vasodilation and the more severe the diabetes, the faster the foot temperature rises. **Limitations:** ignored the effect of ageing and adipose thickness, didn’t consider the multi-layer structure of plantar soft tissues.
Mi et al (2007)	Computational (USA)	Investigate the mechanism of DFU wound healing	A, C5	Blood and tissue (neutrophils, macrophages, and fibroblasts, mediators)	Not validated	**Participants:** not mentioned. **Equipment:** not mentioned. **Boundary conditions:** no experiments.	**Key Findings:** Elevated TNF (tumour necrosis factor) and reduced TGF-β1 (Transforming growth factor-β1) could be two important mechanisms of DFU. **Limitations:** Ignored collagen contraction as part of the wound-healing process.
Sun et al (2009)	Computational (UK)	Investigate the effect of TGF-β1 in epidermis wound healing	A, C5	epidermis	Compared with literature	**Participants:** not mentioned. **Equipment:** not mentioned. **Boundary conditions:** no experiments.	**Key Findings:** Active TGF-β1 has a significant effect on early wound healing, hard-to-heal chronic wounds may be caused by abnormal cell migration or cell proliferation. **Limitations:** only considered the effect of TGF-β1.
Yang et al (2013)	Computational (USA)	Investigate the effect of wound shape and mechanical environment to wound healing	A, C5	skin	Compared with literature	**Participants:** not mentioned. **Equipment:** not mentioned. **Boundary conditions:** no experiments.	**Key Findings:** Determined a novel wound contraction mechanism. **Limitations:** The modelling size is too small, ignores the viscosity of soft tissues, still a 2-dimensional model
Peng et al (2020)	Computational (Netherlands)	Simulation of skin contraction	A, C5	skin	Compared with literature	**Participants:** not mentioned. **Equipment:** not mentioned. **Boundary conditions:** no experiments.	**Key Findings:** Macrophages have a significant effect in the early stage of wound healing. **Limitations:** only a 2-dimensional model

Macroscopic to microscopic: C1: 2D cross sectional model, C2: 3D whole foot model, C3: specific region model, C4: tissue level model, C5: micro-level model.

Different focused factors: M: mechanical stress model, F: vascular and nerve model, P: multiphysics model, T: thermal model, A: agent-based model.

### Mechanical stress modelling of foot structure

#### Geometry definition of mechanical foot modelling.

Due to the complexity of foot tissues and structures, in order to obtain accurate foot structures, many studies [30,32–34,38,39,44–47] have used medical imaging methods such as Magnetic Resonance Imaging (MRI) or Computed Tomography (CT) scans to obtain anatomical information about the foot. This process is time-consuming and requires a great deal of expertise, some studies [32] have chosen to model the foot in two dimensions using a cross-section along the metatarsal rays of the foot, which is adequate for evaluating the pressure distribution under the metatarsals and the relative influence of different tissues on plantar pressure, but the 2D model neglects the spatial effects of pressure and is not able to effectively represent the internal interactions or sliding effects of the skin, fat, muscle, and bone.

Nowadays, more researchers [30,33,34,38,39,44–47] choose to build 3D foot models to comprehensively simulate the characteristics of the foot. Among the nine articles included in this study on 3D mechanical foot models, about half of the models modelled the whole structure of the foot (including the ankle) [33,34,38,39], which has the advantage of comprehensively simulating the effects of different tissues of the foot on plantar pressures, but the computational cost incurred by this kind of model is extremely large, and in 50% of these articles the multilayered structure of the plantar soft tissues is not taken into account to simplify the calculations [33,34]. All these articles modelled the plantar soft tissues as a homogeneous entity. Although this is sufficient for describing the plantar pressure, it cannot represent the effect or damage in deeper plantar tissues.

Over half of the remaining studies modelled specific regions of the foot [30,44–47,49], to avoid the effects of inconsistencies in the thickness of soft tissue structures in different areas, providing a more accurate evaluation of the effects of diabetes on specific areas, and significantly reducing the computational cost.

In recent years, it has been demonstrated that pathophysiological lesions of the plantar soft tissues are one possible cause of DFU [12], and it has been found that diabetes leads to a significant thickening of the dermis, thickening of the elastic septa in the fat pad [60], and stiffening of plantar soft tissues [61].

**Fig 2:**
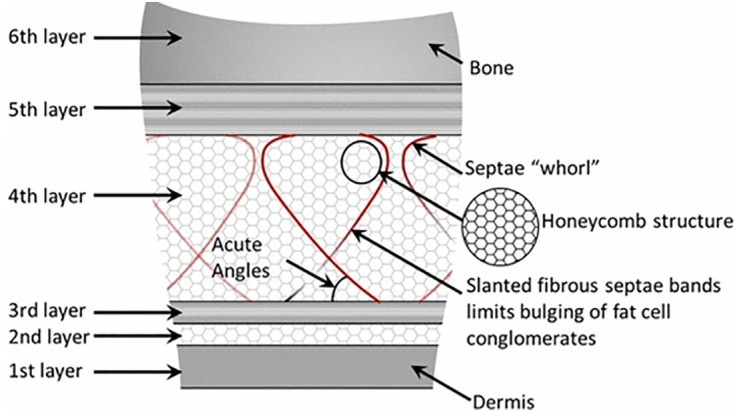
Schematic structure of the plantar soft tissues, obtained from Ou et al. (2021) [51]. First layer: dermis, second layer: superficial subcutaneous, third layer: panniculus carnosus, fourth layer: adipose structure, fifth layer: deeper subcutaneous, sixth layer: bone structure.

In order to have a better investigation of the mechanical behaviour of plantar soft tissues, studies have been undertaken to develop tissue-level foot models [31,50–52,55], which usually do not consider the exact geometry of the whole foot, but rather the microstructure of the plantar soft tissues as shown in Fig 2.

**Fig 3:**
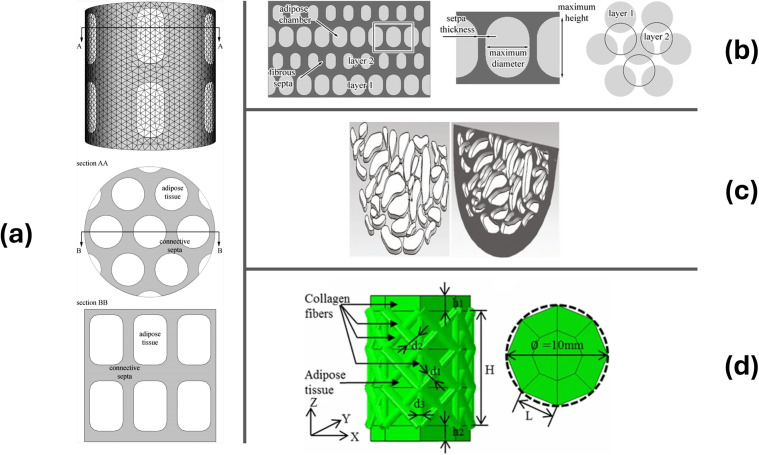
Geometry of plantar adipose structure. **(a)**. fat pad model with chambers. **(b)**. fat pad model with two kinds of chambers. **(c)**. fat pad adipose tissue model. **(d)**. adipose tissue model with collagen fibres, adapted from [31,50,51,55].

Currently, no studies have modelled the complete six-layers of the plantar soft tissue structure, most studies focused on the adipose structure and deeper tissues [31,50,51,55], because researchers have demonstrated that diabetes can lead to the thickening of the connective setae in the plantar adipose structure and irregular alignment of collagen fibres [62]. This can lead to a reduction in the size of the adipose chambers, change the plantar pressure distribution, and cause a stress concentration in the specific areas of the plantar foot. As shown in Fig 3, four of the five articles [31,50,51,55] included in this study on tissue-level models modelled the honeycomb adipose structure and in terms of the shape of the adipose chambers, two studies modelled it as an ellipsoid [31,55], one as an irregularly distributed teardrop [50], and the remaining one as a multivariate [51], where the dimensions of the components of the model are shown in Table 2.

**Table 2:** Geometry parameters of plantar adipose structure.

Parameter	Value	References
Healthy connective septae thickness	95 - 2650 *µm*	[31,51,55]
Degraded connective septae thickness	209–331 *µm*	[55]
Connective septae orientation	0, 15, 30, 45, 90, 135, 180°	[31,51,55]
Max healthy adipose chamber diameter	900 - 3000 *µm*	[31,55]
Max degraded adipose chamber diameter	600 *µm*	[55]
Max healthy adipose chamber height	1250 *µm*	[55]
Max degraded adipose chamber height	800 *µm*	[55]

Anatomical experiments have shown that the fat structure of the heel is different from that of other regions of the foot, consisting of superficial microchambers and deep macrochambers [63] (Fig 3(c)), the different sizes and relative arrangements of the two adipose chambers should be considered during the modelling. This type of model can better investigate the effects of external forces on tissue deformation or damage by observing the interaction of plantar soft tissues under mechanical stress, and models with adipose chambers can better simulate the processes of energy absorption and impact attenuation during the gait cycle [50]. Compared with subject-specific 3D foot models, tissue-level foot models have the potential to provide a generalised understanding of the mechanical behaviour of the diabetic foot. Future studies should develop a complete multilayer microscopic model of the plantar soft tissue and consider the time factor and the effects of tissue damage, to further observe the effects of diabetes on the plantar soft tissue.

#### Mechanical properties of plantar soft tissues.

Currently, it is well demonstrated that plantar soft tissue material properties show non-linear viscoelastic behaviour [64,65], under the influence of diabetes the plantar soft tissues will become thicker and stiffer [38,52]. In this study, the mechanical properties of plantar soft tissues used in previous research are summarized in Table 3.

**Table 3:** Plantar soft tissues mechanical properties range table.

Component	Material Model	Material Properties														Reference
		Material Elastic Properties														
		Young’s Modulus (MPa)		Elastic Modulus (MPa)			Shear Modulus (MPa)		Bulk Modulus (MPa)		Poisson’s Ratio			Mass Density (g/mm^2^)		
Bone (whole structure)	Linear elastic	7300-7900		—			—		—		0.3000			—		[32,33,36,45,47]
Cortical bone	Linear elastic	12000-20000		—			—		—		0.3000			0.00180-0.00200		[39]
Trabecular bone	Linear elastic	7000-7200		—			—		—		0.3000			0.00160		[39]
Skin	Elastic/hyperelastic	0.00170		0.970850			0.3247		32.3570		0.4000-0.4999			0.00092		[45–47]
Fat structure	Hyperelastic/linear elastic	0.03030-0.06120		0.000855			0.000286		0.0285		0.4900-0.4999			0.00100		[44–47,51]
Fibre sturcture	Linear elastic	1.15000		—			—		—		0.4900			—		[50,51]
Muscle	Elastic/visco-hyperelastic	0.00952-1.08000		—			—		—		0.4900-0.4999			0.00100-0.00112		[44,46,47]
Plantar soft tissues	Linear elastic	1.0		—			—		—		0.4000			—		[36]
		**Hyperelastic Material Properties**														
		***µ*(MPa)**	***α***		***C***_***10***_ **(*N/mm*** ^***2***^ **)**	***C***_***20***_ **(*N/mm*** ^***2***^ **)**		***C***_***30***_ **(*N/mm*** ^***2***^ **)**	***C***_***01***_ **(*N/mm*** ^***2***^ **)**	***C***_***02***_ **(*N/mm*** ^***2***^ **)**		***C***_***11***_ **(*N/mm*** ^***2***^ **)**	***D***_***1***_ **(*mm*** ^***2***^ ***/N*)**		***D***_***2***_ **(*mm*** ^***2***^ ***/N*)**	
Skin	First order Odgen	0.4520-0.6400	5.6-6.9		—	—		—	—	—		—	—		—	[44,47]
Skin	Second order polynomial	—	—		0.08600	0.03900		—	−0.05800	0.00900		−0.02300	3.65200		0	[39]
Skin	Yeon law	—	—		0.2650	1.9230		0	—	—		—	—		—	[46]
Fat structure	First order Ogden	0.0032-0.0950	4.50-7.42		—	—		—	—	—		—	—		—	[44,46,47]
Fat structure	Second order polynomial	—	—		0.08556	0.03900		—	−0.05841	0.00851		−0.0231	3.65273		0	[50]
Collagen fibres	Second order polynomial	—	—		5.21	5.08		—	−4.58	0.83		−3.42	0.0319		0	[51]
Muscle	First order Ogden	0.00343-0.05700	8.740-28.82		—	—		—	—	—		—	—		—	[39]
Muscle	Yeon law	—	—		0.0050	0.0690		1.9670	—	—		—	—			[46]
Plantar soft tissues (healthy)	Second order polynomial	—	—		0.00671-0.08556	0.00339-0.03900		—	−0.05841	0.00851		−0.02319	2.42000-3.65273		0	[33,51]
		**Fibre Reinforced Hyperelastic Material Properties**														
		***K***_***v***_ **(MPa)**			***r***			***C***_***1***_ **(MPa)**		***α***_***1***_			***C***_***4***_ **(MPa)**		***α***_***4***_	
Fat structure	hyperelastic	0.231			27.4			0.00219		53.5			—		—	[31,55]
Fibre Septae	hyperelastic	0.0202			—			0.00463		—			0.23600		54.8	[31,55]
Plantar soft tissues (healthy)	hyperelastic	0.0105-0.0400			26.4000-57.1181			0.0013-0.00393		1.6600-3.3338			—		—	[30,34,55]
Plantar soft tissues (degenerative)	hyperelastic	0.125-0.250			13.5-49.5			0.00184-0.0245		1.53-3.66			—		—	[34,55]
Plantar soft tissues (disease)	hyperelastic	0.438			10.5			0.0429		1.19			—		—	[34]
		**Viscous Elastic Material Properties**														
		***G***_***1***_ **(MPa)**	***β***_***1***_ **(ms)**		$\gamma_{1}$	$\tau_{1}$ **(s)**		$\gamma_{2}$	$\tau_{2}$ **(s)**	$\gamma_{3}$		$\tau_{3}$ **(s)**	$\gamma_{4}$		$\tau_{4}$ **(s)**	
Skin	First order Prony series viscoelstic	0.42	0.12		—	—		—	—	—		—	—		—	[44]
Fat structure	Viscoelastic	0.24-0.30	0.12		—	—		—	—	—		—	—		—	[44]
Plantar soft tissues	Visco-hyperelastic	—	—		0.1874-0.7170	0.000623-37.4249		0.1550-0.3682	0.0155-0.0331	0.0652		98800	0.0626		982000	[30,34]

Hyperelastic material coefficient: *µ* relates to the stiffness or strength of the material and determines the response of the material during tension or shear, *α* represents the degree of non-linearity in the stress-strain relationship of the material $C_{ij}$ represents the shear behaviour of the material, *D*_1_ and *D*_2_ demonstrates the volume compression properties of the material.

Fibre-reinforced hyperelastic coefficient: *K*_v_ represents the initial volumetric stiffness, *C*_1_ represents the shear stiffness, *r* and *α* represents the evolution of material stiffness.

Viscous elastic coefficient: *G*_1_ is the elasticity modulus which represents the energy stored in the material during the transient elastic response, *β*_1_ is viscoelastic time constant, larger *β*_1_ means slower relaxation of the material, $\gamma_{j}$scale factor related to the elastic or viscous properties of the material, $\tau_{i}$ usually describes the stress relaxation time of the material or the characteristic time during creep.

In Table 3, the material models used to describe plantar soft tissues were classified into four main categories: linear elastic material model, hyperelastic material model, the fibre-reinforced hyperelastic elastic material model and the viscous material model. In all studies included in this paper, the bony structures are modelled as a linear elastic material [32,33,36,39,44–47,50,51], only a few studies modelled the plantar soft tissue as a linear elastic material, ignoring its nonlinear properties. Although it would reduce the complexity of the calculations, this approach would distort large strains and cannot reflect its hardening effect under the influence of diabetes mellitus, and it would also neglect the stress-strain relationship versus the time.

The plantar soft tissues were modelled using hyperelastic materials in most of the articles, and the commonly used models are Ogden, Yeon and Neo-Hookean models, which are essentially strain-energy density functions to describe the stress behaviour of materials under large strains [66]. Most of the modelling used the Ogden or the polynomial hyperelastic model [33,39,44,47,50,51], which are multi-parameter models that describe the mechanical behaviour of the material [66]. They can be better able to fit the experimental data than the one-parameter Neo-Hookean model, and better able to deal with multiaxial strains than other models [67].

Several studies have also investigated the effect of the loss of plantar elastin and the fracture or damage of the fibrous septa due to ulceration in diabetic mellitus [30,31,34,55]. These studies modelled the plantar soft tissue using a fibre-reinforced hyperelastic model. This approach is significantly different from traditional hyperelastic models, which typically assume that plantar soft tissues are isotropic material [68]. In contrast, the fibre-reinforced hyperelastic model considers the specific arrangement and orientation of elastic fibres within the soft tissue [30,31]. By modelling the tensile response of these fibres separately, this approach enables the capture of non-linear stress-strain relationships in different directions, which provides a more accurate representation of the biomechanical behaviour of the plantar soft tissue under various loading conditions [31,34,55].

Recently, the time-dependent stress response of plantar soft tissue has been increasingly recognized, and the findings indicate that the stress-strain curves of plantar soft tissue during loading and unloading do not overlap, which suggests that the plantar soft tissues exhibit viscoelastic properties [64,69–71]. However, most studies have ignored this characteristic to reduce computational costs, with only around 20% of the literature defining the viscoelasticity of plantar soft tissue during their modelling [30,34,44]. Research has also demonstrated that the viscoelastic properties of plantar soft tissue are significantly elevated in people living with diabetes compared to people living without diabetes and are positively correlated with higher peak pressure gradients (PPG) [69]. Furthermore, a reduction in the viscoelasticity of soft tissue has been associated with diminished cushioning of the foot, with an increase in the risk of ulceration. Therefore, it is crucial to incorporate these viscoelastic properties into the modelling process of plantar soft tissues.

It is important to note that the hyperelastic material coefficients presented in Table 3 have considerable variation across different studies. This variability may be caused by the inherent heterogeneity of soft tissues and inter-individual differences in the sources of experimental materials, but studies have not quantified the effect of differing coefficients on the simulation results.

#### Meshing and boundary conditions of foot modelling.

Most current research used solid tetrahedral or solid hexahedral units for modelling foot bony structures and plantar soft tissues [30,33,34,38,44,46,47,50,55]. Tetrahedral elements are better suited for automated meshing and are often the first choice for developing foot models [72]. Although this type of mesh saves a great deal of computational cost, it is not as accurate as hexahedral elements. Studies have demonstrated that, although both tetrahedral and hexahedral elements can produce smooth and uniform pressure distributions across the plantar foot, the peak contact pressures predicted by linear tetrahedral elements tend to be 20–30% higher than the actual values [72,73]. Additionally, the compressive and shear stresses predicted by linear tetrahedral elements are often noisy and mesh-dependent [73]. In contrast, both quadratic tetrahedral and linear hexahedral elements can accurately represent plantar pressure distributions. Nevertheless, the computational time for quadratic tetrahedral elements is typically two to three times longer than that of linear hexahedral elements under the same conditions [73,74], so the linear hexahedron element is a preferable choice for foot modelling.

In all reviewed articles, the forces applied to the model are mainly classified into ground reaction forces and muscle forces inside the foot. Ground reaction forces are usually related to one’s body weight, and most of the studies applied forces equal to 50% of one’s body weight to the ankle joints to simulate static human standing [33]. Muscle forces within the foot are often used for the six major muscles at the ankle (lateral gastrocnemius, medial gastrocnemius, soleus, tibialis posterior, peroneus longus, and tibialis anterior), which are mostly measured by gait experiments and change continuously as the gait cycle progresses [75,76]. In addition to the vertical forces and their reactions, the lateral shear force on the plantar foot is also an important factor in the boundary conditions. Studies have demonstrated that shear stress plays a role in more than 50% of plantar injuries [45], and diabetic or ulcerated populations tend to have higher shear stresses compared to healthy populations [77], shear stresses are often represented by friction in foot models, with friction coefficients taken at 0.42 to 0.6 [33,34,38,39].

In the tissue-level foot model shown in Fig 3, the lower surface of the model is usually completely fixed to prevent displacement of the model when an external force is applied [31,51,52,55], and the lateral degrees of freedom are unrestricted to more realistically simulate the behaviour of the foot soft tissue under compressive forces [31,55]. Additionally, most studies have assumed the existence of bonded connections between the layers of soft tissue in the foot, thus ignoring the influence of interlayer soft tissue sliding effects [50–52]. However, it has been demonstrated that a model considering soft tissue sliding contact can more accurately reflect the contact pressure distribution, particularly the pressure distribution under the metatarsal bone is improved [39]. The sliding contact between tissues helps translate stress concentration into a more homogeneous load distribution compared to conventional foot models [78]. Nonetheless, fully accounting for the sliding effect between soft tissues significantly increases computational cost, so the use of a shared mesh should be considered for simulating certain foot regions [78].

A limitation of the current boundary conditions in the plantar soft tissue mechanical models is the absence of models that account for potential torsional loadings between soft tissues. Additionally, the impact of various combinations of loadings on foot tissues, and their role in the formation of diabetic ulcerations has not been fully explored.

### Thermal modelling of foot structure

The studies experimentally demonstrated that there is a significant temperature difference between the plantar skin with diabetic ulcerations and the surrounding foot area [79], with the temperature of the foot in the inflamed or infected area being 2.2°C higher than the temperature in the same area of the contralateral foot [80], so it is clear that temperature is an important indicator for monitoring the formation and progression of DFU. Currently, thermometry experiments for DFU patients rely on the use of an infrared temperature detector for direct measurement of foot temperature [41,42].

Three articles identified in this study conducted a 2D cross sectional model [40,42] or a simplified 3D whole foot level model [41] to investigate the temperature changes. The 2D foot model was constructed based on direct physical measurements [42], and the 3D model was derived from 3D scans of actual human feet [41]. One of the studies also modelled the ulcerated areas on the plantar foot surface, these areas were represented as elliptical shapes and positioned on the plane 5 mm beneath the plantar skin, with the material properties assigned to muscle tissues [41].

In all three thermal modelling articles included in this study, the bioheat transfer within the tissue was described using Pennes bioheat equation [40–42], a mathematical framework used to describe the tesmperature distribution in biological tissues, which assumes the tissue is a continuous homogeneous medium and ignores variations in the tissue properties [81]. The representation is shown in Equation 1.


$$\begin{array}{c} \rho c_{p}\frac{\partial T}{\partial t}+\nabla \cdot (-k\nabla T)=\omega_{b}c_{b}\left(T_{b}-T\right)+Q_{met}+Q_{ext} \end{array}$$ (1)

Where ρ is the tissue density $\left[kg/m^{\text{3}}\right]$, $c_{p}$ is the specific heat capacity of the tissue [J/kg·K], T represents the tissue temperature[K], t describes the time [s], k is the thermal conductivity of the tissue [W/m·K], $\omega_{b}\;$is the blood perfusion rate$\;[kg/m^{\text{3}}\cdot s]$, $c_{b}$ is the specific heat capacity of the blood [J/kg·K], $T_{b}$ describes the arterial blood temperature [K], $Q_{met}$ is the metabolism heat$\;[W/m^{\text{3}}]$, and $Q_{ext}$ represents the external heat $\left[W/m^{\text{3}}\right]$.

The parameters set as input in the models are represented in Table 4.

**Table 4:** Pennes bioheat equation parameters for foot heat transfer.

Parameter	Value	Reference(s)
Tissue density $\left[kg/m^{\text{3}}\right]$	1109	[41]
Blood density $\left[kg/m^{\text{3}}\right]$	1000-1060	[41,42]
Tissue specific heat capacity [J/kg·K]	3391	[41]
Blood specific heat capacity [J/kg·K]	3639-3850	[41,42]
Blood perfusion rate $\;[kg/m^{\text{3}}\cdot s]$	0.0036-0.0054	[41,42]
Thermal conductivity of the tissue [W/m·K]	0.3700-0.5000	[41,42]
Thermal conductivity of the skin [W/m·K]	0.2000	[41]
Arterial blood temperature [K]	310.15	[41]
Metabolism heat $\left[W/m^{\text{3}}\right]$	300	[42]

In contrast to the foot mechanical model, the thermodynamic model of the foot needs to consider three boundary conditions. The first one involves heat exchange between the ankle and the adjacent leg segment. However, since the temperature variation among different body parts in the same environment is minimal, this term is typically assumed to be zero in the study [40,42]. The second one addresses heat transfer at the boundary where the soft tissues interface with the external epidermis [42]. If a lossless heat transfer is considered at this interface, the temperature can be treated as continuous across these two regions. The last one is heat transfer which describes the exchange between the skin and the external environment, which accounts for the effects of air and perspiration [40–42]. This heat exchange is typically defined by the parameters provided in Table 5.

**Table 5:** Some typical parameters for skin-environment heat transfer.

Parameter	Value	Reference(s)
Convection coefficient $\left[W/m^{\text{2}}\cdot K\right]$	10	[42]
Skin emissivity	0.97	[42]
Stefan–Boltzman constant $\left[W/m^{\text{2}}\cdot K^{\text{4}}\right]$	5.67$e^{\text{8}}$	[42]
Relative humidity	0.35	[42]

### Vascular and nerve system modelling of foot structure

Abnormal blood flow and neuropathy are also critical factors contributing to DFU. Research has demonstrated that chronic hyperglycaemia leads to reduced vascular permeability and impaired blood flow autoregulation [82]. The atherosclerosis increased peripheral blood flow, and calcification of the arterial intima are also investigated in DFU patients [83,84]. Furthermore, the abnormal distribution of plantar pressures in diabetic patients has a direct impact on the blood vessels [53]. These factors collectively disrupt blood transport and nutrient exchange within tissues, thus impeding the wound healing. Therefore, it is essential to conduct modelling and analysis of blood flow in the vasculature of ulcerated areas in diabetic feet.

Two articles included in this study typically modelled vascular dynamics and foot nerve system independently, without explicitly incorporating the surrounding foot structures or coupling with other DFU-related factors [48,53]. One article [48] employed a graphical programming language to create a physical simulation model of blood vessels, aimed at investigating the effects of plantar pressure, tissue stiffness, and external forces on arterial diameter. In this model, blood was represented as a fluid with properties of inertia, stiffness, and damping. The study found that with increasing external force, both arterial and venous diameters constrict, and the sensitivity of arteries to these forces decreases. This reduction in sensitivity leads to decreased local blood flow in diabetic patients.

The other study modelled blood vessels containing plaques that may cause foot ulceration [53]. A CT scan reconstruction technique was used to build the vessel geometry. In this model, the arterial wall was constructed as a Mooney-Rivlin hyperelastic material, a Yeoh hyperelastic model was used for the modelling of lipid and calcified plaques, and the blood was considered to be a non-Newtonian fluid with a constant density. In terms of boundary conditions, the arterial wall was fixed in degrees of freedom at both ends and was set up as a binding contact with the plaque, with a uniform pressure of 1 kPa applied to the outer surface of the arterial wall. It was demonstrated that low wall shear stress (WSS) may promote the accumulation of new lesions in the vasculature of the foot in diabetic patients, and the oscillating WSS may trigger lesion expansion in healthy areas of the foot.

A comprehensive functional dorsal foot nerve model (CFDNM) for simulating small unmyelinated nerves (SUSSN) and myelinated large nerves (MLN) in the foot [54] was conducted to show the propagation of action potentials through the nerves and calculate nerve conduction velocity (NCV) through image digitisation, path selection algorithms and electrophysiological modelling. The model combines anatomical data and a two-domain model to effectively study nerve conduction mechanisms in the neuropathic foot, which provides a new tool for neuropathy in DFU.

### Multiphysics modelling of foot structure

Diabetes is a complex disease influenced by multiple factors, including a wide range of pathological changes such as vascular damage, neuropathy, and soft tissue stiffening. Conventional single-physics models are often inadequate in capturing the complex interactions across these different domains. Consequently, recent research has focused on developing multi-physics models of the foot that integrate mechanical forces, fluid dynamics, heat transfer, and chemical reactions within cellular matrices [35–37,43]. These models generally represent the foot as an integrated structure and simultaneously consider multiple DFU-related factors, with explicit coupling between different physical or biological processes, which aim to explore the interactions between various physical processes to provide a more comprehensive understanding of the mechanisms behind DFU formation, which can offer deeper insights into the disease and could inform better prevention and treatment strategies.

Four articles identified in this review developed multiphysics models of the foot. Two of these studies focused on solid-fluid interaction models [35,36], capturing soft tissue mechanics and blood flow or tissue media dynamics. One study introduced a comprehensive solid-fluid-neural model, integrating neural function and tissue mechanics [37]. The remaining study presented a liquid-thermal model, examining the temperature regulation within the vascular system of the foot [43].

Among the two solid-liquid models, Mithraratne et al. [35] used the finite element method to model the deformation of plantar soft tissues, coupled with the Navier-Stokes equations to simulate blood flow. The coupling was achieved by the effect of soft tissue deformation on the cross-sectional area of the artery: the hydrostatic pressure generated by the deformation of the soft tissues affecting the vascular wall, thus changing the transport of the blood flow. The geometric relationship between the soft tissue and the artery is updated dynamically based on the deformation state derived from the solution, which enables the spatial position of the artery to change with the deformation of the soft tissue [35]. The effect of different soft tissue stiffness on arterial blood flow was finally explored through numerical simulations.

On the other hand, Sciumè et al. [36] modelled the plantar soft tissue as a porous medium consisting of a solid phase (comprising tissue cells and their extracellular matrix) and a liquid phase (comprising interstitial fluid and dissolved chemical species), and the thermodynamic constrained averaging theory (TCAT) was used to describe the interaction of the solids and fluids and the fluid flow was modelled by Darcy’s law [36]. The coupling is achieved through the transfer of stresses between the solid and the fluid. In this process, tissue deformation not only influences interstitial fluid flow but also affects the transport of oxygen and nutrients via an effective diffusion coefficient.

These two studies of solid-liquid coupled foot models illustrate that diabetes not only changes the mechanical properties of plantar soft tissues and plantar pressure distributions but also affects the hemodynamic of blood flow in the foot through changes in tissue stiffness [35]. Additionally, changes in soft tissue stiffness impact the transport of oxygen and nutrients within the plantar tissue [36]. Studies [35,36] have demonstrated that as plantar pressure increases, deformation of the soft tissues leads to compression of the arterial vasculature, which reduces the ability of blood flow to be transported. Stiffer soft tissues are more likely to cause deformation of the arteries, which exacerbates the obstruction of blood flow [35]. At the same time, tissue stress and deformation lead to complex interactions between the liquid and solid phases of the soft tissues, which affects the health of the tissue and accelerates DFU formation.

**Fig 4:**
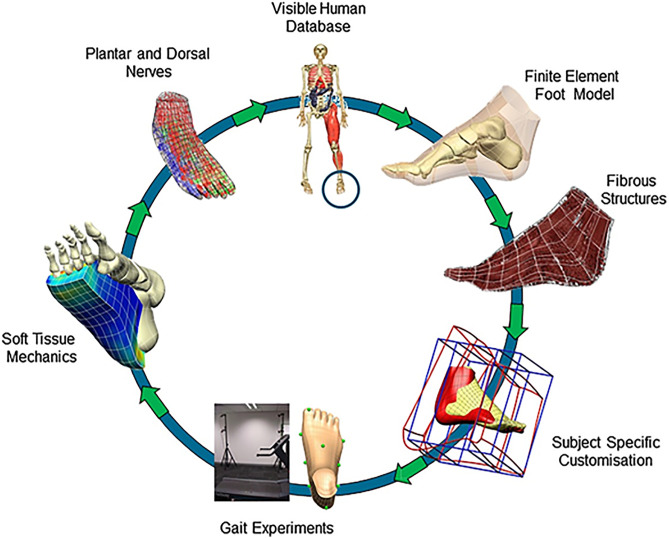
The modelling process of the solid-nerve model, taken from [37].

An additional study [37] established a coupled solid-phase nerve model. As shown in Fig 4, similar to the solid-liquid model, the model is also based on the soft tissue model of the foot achieved by the finite element method, the distribution and growth of the nerves were simulated by the branching algorithm, and the branching nerves are embedded in the soft tissue model [37]. The model coupled the stress deformation of the soft tissue with the interactions of the nerve conduction and blood flow systems. It was found that with the increase of plantar pressure, the stress generated in the soft tissue exerted additional compression on the nerve fibres, which led to a change in the geometry of the nerve fibres, affecting the rate of arrival and efficiency of the action potential [37].

Thus, research has already demonstrated that pressure changes within plantar soft tissue affect both blood flow and the nerve system. However, few studies have explored their interactions with temperature. The work by Wang et al. [43] incorporates the impact of blood flow variations on heat transfer and brings this important relationship into consideration. The model describes the heat transfer between blood flow and surrounding tissues using a heat conduction and convection model, which couples changes in vessel diameter with temperature changes between tissues to analyse the overall heat distribution in the foot. The study demonstrated that in the vicinity of the large blood vessels, the skin temperature is higher, and the skin temperature of the plantar foot shows greater abnormality when the anterior tibial artery is blocked. In people living with diabetes, skin temperature rises with increased blood input. The more severe the diabetes, the faster the skin temperature rises and the more difficult it is to return the foot temperature to its original level after heating [43].

### Cellular level modelling of skin wound or diabetic foot

The formation of DFU and the wound-healing process is a highly complex biological phenomenon. While macroscopic foot models can help investigate the effects of physical factors such as pressure, temperature, and blood flow on ulceration formation and their interactions, these models are insufficient to explain the pathological mechanisms of DFU formation. To fully understand the development and healing mechanisms of diabetic foot ulcers, it is essential to investigate cellular behaviours on the plantar foot. Changes at the cellular level, particularly the roles of various cell types during inflammation, regeneration, and tissue repair, are crucial for describing the microscopic healing mechanisms of wounds. Studying the dynamic behaviour of these cells throughout the different stages of wound healing can provide insights into the complex pathology of DFUs and offer a theoretical basis for developing effective healing strategies.

Four articles included in this study discussed the cellular behaviours of the skin wound repair or diabetic foot healing [26,27,57–59], and all articles modelled the cells on the skin through agent-based modelling method [26,57–59]. Agent-based modelling is a computational simulation method that can be used to study individual cell behaviour and their interactions in complex biological systems and is currently being used in some articles for the study of skin wound healing [26,57] and cancer spread simulation [85].

Agent-based models consist of three key elements: agents, the environment and patches [26]. In biological cell simulations, the environment is usually represented as the extracellular matrix (ECM), agents represent different types of cells, and patches correspond to various mediators and growth factors secreted by the cells. These patches not only affect cell behaviour but also regulate cell generation and development. Because the healing process of diabetic foot ulcer wounds is divided into four phases: coagulation, inflammation, proliferation and remodelling [59], studies usually focus on modelling the following six types of cells: fibroblasts and myofibroblasts, keratinocytes, macrophages, neutrophils and endothelial cells [26,27,57–59].

During the early stages of wound healing platelets aggregate at the wound site to form a clot, while platelet-derived growth factor (PDGF) is released to signal subsequent cell migration and tissue repair [57]. Neutrophils and macrophages then enter the wound area to remove necrotic tissue and pathogens, and macrophages also secrete tumour necrosis factor *α*(TNF-*α*) and interleukins (ILs) to regulate the inflammatory response of the tissue [26]. During the proliferation phase, fibroblasts secrete collagen to form a new ECM, and keratinocytes migrate from the wound edges until they cover the wound and form a new epidermal layer [26,57]. Throughout this process, both macrophages and fibroblasts also secrete transforming growth factors *β*(TGF-*β*), which aids fibroblast activation and collagen deposition [26]. Finally, in the remodelling phase, matrix metalloproteinases (MMPs) regulate the dynamic equilibrium by degrading the excess collagen, and the collagen fibres are rearranged evenly [59].

Research has shown that fibroblasts provide essential structural support during wound repair, and macrophages promote the inflammatory response by secreting pro-inflammatory cytokines [26,57]. However, in the formation of DFU, this inflammatory response is often excessively activated [26], resulting in chronic inflammation. Diabetic patients typically exhibit elevated levels of TNF-*α* and reduced levels of TGF-β [26], which results in the inhibition of normal tissue repair and makes wound healing difficult. Additionally, studies have found that MMPs are abnormally expressed in diabetic patients [59], leading to excessive tissue degradation, which further impairs the healing process.

## Discussion

Currently, mechanical stress foot models have become more mature, both 2D or 3D whole-foot models or tissue-level microstructural models can accurately simulate the distribution of plantar pressure. Through mechanical stress modelling of foot biomechanics, it can be found that the pressure distribution of the plantar foot is affected by material properties of bones, tendons, fascia and soft tissues working together to determine the pressure distribution in this region [32].

The findings indicate that the interface between plantar soft tissue and irregular bony structures generates elevated von Mises stress [74], corroborating prior research that localises peak plantar pressure within the heel region [47,49,78]. This pressure concentration likely contributes to the high incidence of DFU in the metatarsal and heel areas. Additionally, the multilayered plantar soft tissue structure analysis reveals that adipose tissue exhibits high sensitivity to stress, with a significantly lower injury threshold than the skin [45]. This vulnerability is exacerbated by diabetes-induced degeneration of adipose tissue, which results in increased stiffness and a diminished capacity for energy absorption [34,46,51,52,55]. Consequently, stress concentrations at these sites are intensified, supporting the hypothesis that DFU originate in the deeper subcutaneous tissues rather than at the epidermal surface [47].

Secondly, it was found that 50% of the effective stress for DFU is caused by plantar shear stress in both the skin and adipose layers [45]. This finding suggests that friction during walking is a significant contributor to the formation of DFU [45]. Age and gender related tissue stiffness also emerge as significant factors, with the stiffness of the plantar soft tissues at heel increasing with age, and the metatarsal tissue being marginally stiffer and thicker in males than in females [52,86]. This suggests that individual variability substantially impacts plantar pressure distribution.

However, while mechanical stress models can reveal the influence of mechanical factors during ulcer formation, they still have certain limitations. For instance, most of the current biomechanical models of the foot [30,33,34,38,39,44–47] predominantly rely on medical imaging from a single subject, which limits their ability to capture the anatomical variability that exists across populations, including differences in plantar soft tissue thickness, skeletal alignment, and diabetes-related structural changes. As a result, these models have limited capacity to quantify the relationship between anatomical variation and the risk of DFU development, which in turn reduces their potential value for personalized ulceration risk assessment.

In addition, mechanical stress models mostly ignore the sliding effects between the layers of plantar soft tissue [30,33,34,38,44–47], as well as the changes in soft tissue material properties over time [87], and most of these models will use a von Mises failure criteria which is not a good fit for soft tissue failure. Other studies related to the mechanics of soft tissues use failure criteria such as Tsai Wu [88] which is likely to more closely match tissue failure and breakdown. These simplifications may lead to an underestimation of stress concentrations in deep tissues, because deep tissue damage often develops prior to visible skin breakdown, the inability to accurately model deep tissue stress concentrations and failure mechanisms may limit the clinical applicability of these models for early ulceration prediction, thereby affecting the design of preventive interventions such as custom insoles and other offloading strategies.

In terms of thermal foot models, the studies found that the lowest temperature on the plantar surface is located at the toes [42], and the temperature at the ulceration site is significantly higher than at other plantar foot locations. Additionally, the temperature at the ulceration site does not dissipate over time, and it is not influenced by the surrounding air temperature [41]. The DFU area also radiates heat to the surrounding tissues, which leads to an overall increase in temperature across the foot. This heat transfer exhibits a distinctly nonlinear characteristic, which shows that the elevated lower limb temperature observed in people living with diabetes may be attributed to the heat dissipation from the ulceration site [41]. However, it can be noticed that all three studies only focused on the external contours of the foot and ignored the vascular network, the internal bone structures and the multiple layers of plantar soft tissue [40–42]. The models treated the internal components of the foot as a homogeneous material, with the skin being the only anatomical feature modelled separately.

Both the blood flow model and the nerve model of the foot offer new perspectives for understanding the formation of DFU [48,53,54]. The research found that the hardening of plantar soft tissues in diabetic patients makes blood vessels more susceptible to pressure change, resulting in a more severe impairment of blood flow [48]. Additionally, areas of high stress at the shoulder of the plaque are associated with the risk of plaque rupture [53], which has similarities to vascular fragility and local ischaemia in DFUs. Calcified plaques may reduce the risk of rupture in some plaques but may also lead to the accumulation of new plaques and aggravate the condition.

However, there are limitations that standalone vascular or nervous system models are insufficient to fully explain the complex mechanisms of DFU. These models only objectively describe the effects of diabetes on blood flow or nerves [48,53,54]. To further this research, it would be beneficial to integrate blood flow or nerve models with tissue-level mechanical models of stress and thermodynamics to form multiphysics models that provide a better understanding of the interaction of these mechanisms.

Multiphysics models of the diabetic foot can combine multiple physical processes such as mechanics, haemodynamics, nerve conduction and heat conduction. These models can provide a deeper understanding of the interactions between plantar pressure, blood flow changes, and nerve damage and offer a novel perspective for research on the prevention and treatment of diabetic foot. However, current models are still limited by a simplification of tissue structures and not linking all the physical mechanisms (mechanical stress, thermal, blood flow, etc) into one model.

Agent-based cellular models have looked at inflammatory responses, angiogenesis and skin regeneration [27,56–59]. However, there is still further work required to model complex cellular behaviours accurately and link these with other multiphysics approaches. Other studies have applied the mechanobiological modelling approaches to bone healing [89] and vascular repair [90], but no study has used this method on plantar tissue or DFU.

Overall, a well-validated, multiphysics, multilayer foot model that can realistically represent interactions between soft tissue layers, time-dependent material behaviour, and tissue failure mechanisms would have important clinical value. Such a model could enable more accurate identification of high-risk regions and patient-specific ulceration development mechanisms, while also providing clinicians with quantitative guidance to optimize personalized offloading strategies, monitor disease progression, and evaluate the effectiveness of interventions. Consequently, future research should focus on improving existing models by incorporating larger and more diverse subject cohorts, accounting for multiple DFU-related influencing factors, and integrating more realistic representations of soft tissue mechanics and failure behaviour, thereby strengthening their potential for clinical translation in the prediction and prevention of DFU.

### Strengths and limitations

This study has several important strengths. First, this is the first scoping review to systematically examine a wide range of foot models, encompassing different modelling strategies and model types. Based on a comprehensive review of existing literature, this review not only summarizes the current state of development of various foot models but also provides an in-depth analysis of their modelling approaches, application contexts, and remaining limitations. It highlights the potential of computational modelling techniques to advance understanding the mechanisms of DFU, provides a clear overall framework for research progress in this field, and helps identify potential directions for future research.

However, this review still has some limitations. First, the literature search was conducted up to March 2025, and therefore studies published after were not included. Second, the search was restricted to three literature databases and to accessible publications written in English, introducing the possibility of language and selection bias. In addition, this review focused primarily on studies involving computational modelling and did not include purely clinical trials or experimental studies. Finally, no formal quality appraisal of the included studies was performed during the review process.

## Conclusion

This review provides an insight into diabetic foot computational models using mechanical stress, thermal, vascular, nerve, multiphysics and biological approaches. The mechanical stress models are well developed in the literature, but the other approaches need further research. Mechanical stress computational models use finite element methods which replicate the viscoelastic nature of the bulk properties of planter tissue well. However, they don’t separate the multiple tissue layers or use appropriate failure criteria in their analysis which may uncover further insights into DFU causation. Future work should focus on combining mechanical stress, thermal and biological approaches into Multiphysics and/or mechanobiological models to further develop understanding of the diabetic foot.

## Supporting information

S1 File PRISMA-ScR-Fillable-Checklist_10Sept2019. (DOCX)
