# Supplementary material for: A scoping review of computational models of the diabetic foot
Source: PLoS One. 2026 Jun 15;21(6):e0351638. doi: 10.1371/journal.pone.0351638 (PMC13268164; doi:10.1371/journal.pone.0351638)
Supplement: Attachment — Submitted filename: Response to Reviewers.docx [file pone.0351638.s003.docx]

**Response Letter**

All page number in this document refer to the page number in the main manuscript

**Reviewer #1:**

**Comment 1(1):** Scope and Search Strategy: The justification for the exclusion criteria, particularly the broad exclusion of studies from fields like "medicine, pharmacology, veterinary, chemistry…" requires clarification. While the intent to focus on engineering and computational aspects is clear, this blanket exclusion might omit some biomechanical studies published in medical journals. Please refine or better justify these criteria to ensure key interdisciplinary work is captured.

**Response 1(1):** *We thank the reviewer for this important comment and for highlighting the potential ambiguity in the description of the exclusion criteria. We agree that the original wording may have given the impression of a blanket exclusion based on journal subject area.*

*We would like to clarify that our intention was not to exclude studies solely based on their disciplinary field or publication venue. Rather, articles were excluded only when they were primarily clinical, pharmacological, or experimental in nature and did not involve computational modelling, biomechanical analysis, or engineering-based approaches relevant to the study of DFU. Interdisciplinary studies published in medical or clinical journals were carefully screened and retained if they included modelling components aligned with the scope of this review.*

*To avoid misunderstanding, we have revised the wording of the exclusion criteria in the manuscript to more clearly reflect this content-based selection strategy.*

*Please see Section “Methods” – Page 10, please see the text below:*

*“Articles primarily focused on clinical medicine, pharmacology, veterinary science, chemistry, nursing, environmental science, social sciences, decision sciences, or arts and humanities were excluded when they did not involve computational modelling, biomechanical analysis, or engineering-based approaches relevant to DFU.”*

**Comment 1(2):** Additionally, the search strategy is comprehensive, but including terms like "diabetic foot syndrome," "plantar pressure," or "tissue mechanics" could further optimize recall.

**Response 1(2):** *We thank the reviewer for this helpful suggestion. In our original search strategy, the terms “diabetic” and “plantar” were already included in the Web of Science and Scopus searches, which would have captured terms such as “diabetic foot syndrome” and “plantar pressure.” However, we acknowledge that specific terms related to tissue mechanics were not explicitly included. In response, we have updated the search strategy in Web of Science and Scopus to include the additional terms “plantar tissue mechanics” and “foot tissue mechanics.” For PubMed, a comprehensive set of MeSH terms was used, which should already encompass these concepts. The updated search yielded 43 additional records, of which one study was identified as relevant after title and abstract screening and has now been included in the review.*

*Please see Section “Methods” – Page 7- Page 9 for the Search strategy revision, please see the text below:*

*“A scientific literature search was conducted in three databases Web of Science, Scopus and PubMed around computational modelling of foot or plantar tissues. The following search terms were used in three databases, due to differences in indexing systems across databases, the specific search terms vary slightly between the three databases; however, all search strategies were developed based on a consistent underlying logic and conceptual framework to ensure comparability and comprehensiveness of the search:*

*1.	Web of Science: ((TS=(plantar) OR TS=(plantar foot) OR TS=(foot sole) OR TS=(plantar soft tissue*) OR TS=(plantar skin) OR TS=(foot skin) OR TS=(diabetic foot) OR TS=(diabetic feet) OR TS=(neuropathic foot) OR TS=(diabetic foot ulcer*) OR TS=(foot ulcer*) OR TS=(diabetic soft tissue*) OR TS=(plantar tissue mechanic*) OR TS=(foot tissue mechanic*))*

*AND*

*((TS=(finite element*) OR TS=(finite element model*) OR TS=(FE model*) OR TS=(FE analysis*) OR TS=(FEA) OR TS=(FEM) OR TS=(Mechanobiological model*) OR TS=(constitutive model*) OR TS=(computational model*) OR TS=(computational model*) OR TS=(fluid dynamic*) OR TS=(CFD) OR TS=(fluid model*) OR TS=(two phase model*) OR TS=(multi-phase model*) OR TS=(thermodynamic model*) OR TS=(thermal model*) OR TS=(temperature model*) OR TS=(multiphysics model*) OR TS=(computational fluid dynamics)) OR ((TS=(diabetic foot) OR TS=(skin wound)) AND TS=(agent based model*)))*

*NOT*

*(TS = (insole*) OR TS = (shoe*) OR TS = (footwear*) OR TS = (device*) OR TS = (sensor*) OR TS = (deformity) OR TS = (hammer toe*) OR TS = (flatfoot) OR TS = (ostrich foot) OR TS = (amputation) OR TS = (machine learning) OR TS = (deep learning)).*

*2.	Scopus: ((TITLE-ABS-KEY("plantar" OR "plantar foot" OR "foot sole" OR "plantar soft tissue*" OR "plantar skin" OR "foot skin" OR "diabetic foot" OR "diabetic feet" OR "neuropathic foot" OR "diabetic foot ulcer*" OR "foot ulcer*" OR "diabetic soft tissue*" OR "plantar tissue mechanic*" OR "foot tissue mechanic*))*

*AND*

*(TITLE-ABS-KEY("finite element*" OR "finite element model*" OR "FE model*" OR "FE analysis*" OR "FEA" OR "FEM" OR "mechanobiological model*" OR "constitutive model*" OR "computational model*" OR "fluid dynamic*" OR “CFD” OR "fluid model*" OR "two phase model*" OR "multi phase model*" OR "thermodynamic model*" OR "thermal model*" OR "multiphysics model*" OR "blood flow model*" OR "computational fluid dynamics"))) OR ( TITLE-ABS-KEY ( "diabetic foot" OR "skin wound*" ) AND ( TITLE-ABS-KEY ( "agent based model*" )))*

*AND NOT*

*( TITLE-ABS-KEY ( insole* ) OR TITLE-ABS-KEY ( shoe* ) OR TITLE-ABS-KEY ( footwear* ) OR TITLE-ABS-KEY ( device* ) OR TITLE-ABS-KEY ( sensor* ) OR TITLE-ABS-KEY ( deformity ) OR TITLE-ABS-KEY ( hammer AND toe* ) OR TITLE-ABS-KEY ( flatfoot ) OR TITLE-ABS-KEY ( ostrich AND foot ) OR TITLE-ABS-KEY ( amputation ) OR TITLE-ABS-KEY ( deep learning ) OR TITLE-ABS-KEY ( machine learning ))*

*3.	PubMed: (("foot"[MeSH Terms] OR "foot"[All Fields]) AND ("diabetes mellitus"[MeSH Terms] OR ("diabetes"[All Fields] AND "mellitus"[All Fields]) OR "diabetes mellitus"[All Fields] OR "diabetes"[All Fields] OR "diabetic"[All Fields])*

*AND*

*((“finite”[All Fields] AND ("elements"[MeSH Terms] OR "elements"[All Fields] OR "element"[All Fields])) OR ((model[All Fields] OR modeling[All Fields]) AND ("mechanobiological"[All Fields] OR "constitutive"[All Fields] OR "fluid"[All Fields] OR "computational"[All Fields] OR "two phases" OR "three phases"[All Fields] OR "thermodynamic"[All Fields] OR "thermal"[All Fields] OR "multiphysics" OR "blood flow"[All Fields] OR "temperature"[All Fields])))) OR (("diabetic foot"[All Fields] OR "skin wound"[All Fields]) AND ("agent based"[All Fields]))*

*NOT*

*("insole"[All Fields] OR "shoe"[All Fields] OR "footwear"[All Fields] OR "device"[All Fields] OR "sensor"[All Fields] OR "deformity"[All Fields] OR "hammer toe"[All Fields] OR "flatfoot"[All Fields] OR "ostrich foot"[All Fields] OR "amputation"[All Fields] OR "deep learning"[All Fields] OR "machine learning"[All Fields]).”*

**Comment 2:** Please provide further explanation to clarify the distinction between "vascular and nerve system models" and "multiphysics models." For instance, some models categorized as "multiphysics" (e.g., Mithraratne et al., 2012) also involve substantial vascular/blood flow analysis.

**Response 2:** *We thank the reviewer for this valuable comment and for highlighting the potential overlap between vascular-related models and multiphysics models. We agree that some multiphysics studies, such as Mithraratne et al. (2012), include substantial vascular or blood flow analyses.*

*In this review, the distinction between “vascular and nerve system models” and “multiphysics models” is not based on the presence or absence of vascular components alone. Rather, vascular and nerve system models are defined as studies that primarily model blood vessels and/or nerves in isolation, without explicitly incorporating the surrounding foot structures or coupling with other DFU-related factors. In contrast, multiphysics models represent the foot as an integrated structure and simultaneously consider multiple DFU-related factors, with explicit coupling between different physical, biological, or physiological processes.*

*We have added clarifying statements to the manuscript to make this distinction more explicit and to avoid potential confusion regarding models that include vascular analyses within a broader multiphysics framework.*

*Please see Section “Results – Vascular and nerve system modelling of foot structure” – Page 36, please see the text below for the modified description of vascular and nerve system modelling:*

*“Two articles included in this study typically modelled vascular dynamics and foot nerve system independently, without explicitly incorporating the surrounding foot structures or coupling with other DFU-related factors.”*

*Please see Section “Results – Multiphysics modelling of foot structure” – Page 37, please see the text below for the modified description of multiphysics modelling:*

*“These models generally represent the foot as an integrated structure and simultaneously consider multiple DFU-related factors, with explicit coupling between different physical or biological processes.”*

**Comment 3:** To enhance the utility of the summary table (Table 1), it is recommended to add a column for "Primary Research Theme/Objective" for each study.

Furthermore, a brief indication of the model's validation status (e.g., "experimentally validated," "compared with literature," "not validated") in the same table or in the analysis would provide valuable insight into the methodological rigor of the field.

**Response 3:** *We thank the reviewer for this helpful suggestion to improve the clarity and usefulness of the summary table. In response, we have revised Table 1 by adding a new column titled “Primary Research Theme/Objective” for each included study. In addition, we have provided a brief indication of the validation status of each model (e.g., experimentally validated, compared with literature, or not validated) to better reflect the methodological rigor of the existing work.*

*We believe that these additions enhance the interpretability of the review and provide readers with a clearer overview of both the research focus and validation practices within the field.*

*Please see the example rows below, for the full table see main manuscript Section “Results” – Page 15, Table 1.*

**Comment 4:** Please expand the Discussion section to specifically address the implications of these limitations for clinical prediction and prevention. For example, how might the inability to model deep tissue stress concentrations or failure hinder the development of personalized ulcer risk assessment tools? What would be the potential clinical value of a validated, multi-layer model with accurate failure mechanics?

**Response 4:** *We thank the reviewer for this valuable comment. The Discussion has been revised to explicitly address how the limitations of current mechanical stress models may hinder their translation into tools for clinical prediction and prevention. In particular, we discuss the impact of limited anatomical representation and simplified tissue mechanics on personalized diabetic foot ulcer risk assessment, as well as the importance of accurately modelling deep tissue stress concentrations and failure mechanisms. The potential clinical value of a validated multilayer model with realistic tissue interactions and failure mechanics is also highlighted.*

*Please see Section “Discussions” – Page 43 & 45, please see the revised text below:*

*“However, while mechanical stress models can reveal the influence of mechanical factors during ulcer formation, they still have certain limitations. For instance, most of the current biomechanical models of the foot [30,33,34,38,39,44–47] predominantly rely on medical imaging from a single subject, which limits their ability to capture the anatomical variability that exists across populations, including differences in plantar soft tissue thickness, skeletal alignment, and diabetes-related structural changes. As a result, these models have limited capacity to quantify the relationship between anatomical variation and the risk of DFU development, which in turn reduces their potential value for personalized ulceration risk assessment.*

*In addition, mechanical stress models mostly ignore the sliding effects between the layers of plantar soft tissue [30,33,34,38,44–47], as well as the changes in soft tissue material properties over time [87], and most of these models will use a von Mises failure criteria which is not a good fit for soft tissue failure. Other studies related to the mechanics of soft tissues use failure criteria such as Tsai Wu [88] which is likely to more closely match tissue failure and breakdown. These simplifications may lead to an underestimation of stress concentrations in deep tissues, because deep tissue damage often develops prior to visible skin breakdown, the inability to accurately model deep tissue stress concentrations and failure mechanisms may limit the clinical applicability of these models for early ulceration prediction, thereby affecting the design of preventive interventions such as custom insoles and other offloading strategies.”*

*“Overall, a well-validated, multiphysics, multilayer foot model that can realistically represent interactions between soft tissue layers, time-dependent material behaviour, and tissue failure mechanisms would have important clinical value. Such a model could enable more accurate identification of high-risk regions and patient-specific ulceration development mechanisms, while also providing clinicians with quantitative guidance to optimize personalized offloading strategies, monitor disease progression, and evaluate the effectiveness of interventions. Consequently, future research should focus on improving existing models by incorporating larger and more diverse subject cohorts, accounting for multiple DFU-related influencing factors, and integrating more realistic representations of soft tissue mechanics and failure behaviour, thereby strengthening their potential for clinical translation in the prediction and prevention of DFU.”*

**Comment 5:** P2, Abstract: "couple these together to to develop" – remove duplicate "to"

**Response 5:** *Thank you for pointing out this typographical error. We have corrected the duplicate word “to” in the Abstract (Page 2).*

**Comment 6:** P5: "objectivities" should likely be "objectives"

**Response 6:** *Thank you for pointing out this typographical error. We have corrected the word in the Introduction (Page 6).*

**Reviewer #2:**

**Comment 1:** Introduction: I consider the Introduction to be appropriate in terms of contextualizing diabetic foot as a pathology in the international setting and highlighting its clinical relevance. However, it is not sufficiently informative regarding the main types of computational models that have been used to study this condition, nor the existing discrepancies or limitations of these models to date. As I understand it, clarifying these issues is precisely one of the aims of your review.

For example, you later use a classification of models based on “different focused factors” or according to whether they range from “macroscopic to microscopic”. These criteria should be introduced and briefly explained in the Introduction (or at least clearly defined before the Results), so that readers can fully understand and correctly interpret the subsequent classification and findings.

**Response 1:** *We sincerely thank the reviewer for this insightful and constructive comment. We agree that, in the original version, the Introduction did not sufficiently clarify the main types of computational models used to study DFU, nor the rationale behind the classification adopted later in the manuscript.*

*In response to this suggestion, we have revised the Introduction by adding a new paragraph that briefly explains the existing differences and limitations of current computational models in terms of their focused factors, modeling scales, and underlying assumptions. In addition, we explicitly introduce the two complementary classification criteria used in this review—namely, classification based on the dominant factors emphasized by the models and classification based on their spatial modeling scale—without going into detailed descriptions, which are presented in the subsequent sections.*

*We believe that this revision improves the clarity of the manuscript and helps readers better understand and interpret the structure and findings of the review from the outset. The relevant changes have been incorporated into the Introduction section.*

*Please see Section “Introduction – Key investigated factors in DFU and modelling methods” – Page 5, please see the text below:*

*“Although the computational models have contributed to the understanding of the development and progression of DFU from different perspectives, existing studies still show substantial differences and limitations in terms of the factors considered, modelling scales, and underlying assumptions. On the one hand, many models focus on only a single DFU influencing factor, such as mechanical stress, temperature changes, or blood flow, which makes it difficult to fully represent the complex and multifactorial nature of DFU. On the other hand, the spatial scales of these models vary widely, which range from the macroscopic structure of the foot to microscopic tissue and even cellular levels, and clear links between models at different scales are still lacking. Therefore, to systematically review existing studies and clarify their applicability and limitations, this review organizes DFU-related computational models along two complementary dimensions. The first dimension is based on the main biomechanical or biological processes emphasized by the model, which reflects the dominant factors considered in DFU research. The second dimension is based on the spatial scale of the model, distinguishing different levels of representation from the whole foot to local tissue regions and cellular scales. This classification helps highlight differences in research focus and modelling assumptions among existing studies and provides a structured framework for the comparative analysis.”*

**Comment 2:** Methods: The authors should review and implement the PRISMA-ScR recommendations for scoping reviews. I highlight below several aspects of the methods section that need to be improved or clarified:

(1) The guiding research question that underpins the review should be stated explicitly, and, if possible, the framework used to formulate it should be indicated (e.g. PICO, PEO, PECO, etc.).
(2) It should be clearly specified which search terms were MeSH (or equivalent controlled vocabulary) and which were free-text terms. The full search strategies (search equations) for each database, including the exact combinations of terms and Boolean operators, as well as the date limits and final search dates for each database, should be reported.
(3) The number of reviewers involved in each stage of the process should be specified, together with their roles. It should be made clear whether study selection and data extraction were performed in pairs (independently and in duplicate) and how discrepancies were resolved (e.g. consensus, third reviewer). Please also indicate whether any software or assisted systems were used to support these processes. The snowballing procedure that you mention should likewise be described in greater detail (how it was conducted, which references were followed, selection rules, etc.). All flow information (study selection process) should be reported in the Results section, not in the Methods.

At no point is the study selection process clearly explained. It should be specified whether records were first screened by title and abstract and then assessed in full text, and according to which criteria. It is also not clear which variables were extracted from each included study, whether a pre-defined data extraction form was used, and how the data extraction by pairs (if applicable) was organised.

As this is a scoping review, it is acceptable not to perform a formal quality or risk-of-bias assessment of the included studies, although this is obviously a limitation that should be acknowledged. In any case, the Methods should explain how the expected findings will be organised and synthesised (e.g. thematic areas, types of studies to be included, types of models, outcomes, etc.).

In summary, as mentioned earlier, I strongly recommend consulting and adhering to PRISMA-ScR guidelines for this type of review and ensuring that all core methodological elements are reported accordingly.

**Response 2:** *Thank you very much for your detailed and constructive comments. We appreciate your recommendation to adhere more closely to PRISMA-ScR guidelines, which has helped us substantially improve the methodological clarity of our manuscript.*

*In response, we have carefully revised the Methods section in accordance with PRISMA-ScR recommendations and addressed the points raised as follows:*

- ***Guiding research question:*** *We have now explicitly stated the guiding research question in the Aim & Objectives section as research questions, following PRISMA-ScR guidance and relevant exemplary studies.*

*Please see Section “Introduction – Aim and Objectives” – Page 6 for the* ***Guiding research question*** *revision, please see the text below:*

*“The following research question was formulated: What types of computational models have been developed for DFU, what DFU-related mechanisms do they capture, and how do these models contribute to clinical understanding and potential solutions, while highlighting the current state and limitations of the field?”*

- ***Search strategy:*** *We have clarified which search terms correspond to controlled vocabulary (e.g., MeSH) and which are free-text terms. In addition, we have now provided the full search strategies for each database, including Boolean operators, term combinations. Due to differences in indexing systems across databases, the specific search terms vary slightly between the three databases; however, all search strategies were developed based on a consistent underlying logic and conceptual framework to ensure comparability and comprehensiveness of the search.*

*Please see Section “Methods” – Page 7- Page 9 for the Search strategy revision, please see the text below:*

*“A scientific literature search was conducted in three databases Web of Science, Scopus and PubMed around computational modelling of foot or plantar tissues. The following search terms were used in three databases, due to differences in indexing systems across databases, the specific search terms vary slightly between the three databases; however, all search strategies were developed based on a consistent underlying logic and conceptual framework to ensure comparability and comprehensiveness of the search:*

*1.*	***Web of Science:*** *((TS=(plantar) OR TS=(plantar foot) OR TS=(foot sole) OR TS=(plantar soft tissue*) OR TS=(plantar skin) OR TS=(foot skin) OR TS=(diabetic foot) OR TS=(diabetic feet) OR TS=(neuropathic foot) OR TS=(diabetic foot ulcer*) OR TS=(foot ulcer*) OR TS=(diabetic soft tissue*) OR TS=(plantar tissue mechanic*) OR TS=(foot tissue mechanic*))*

*AND*

*((TS=(finite element*) OR TS=(finite element model*) OR TS=(FE model*) OR TS=(FE analysis*) OR TS=(FEA) OR TS=(FEM) OR TS=(Mechanobiological model*) OR TS=(constitutive model*) OR TS=(computational model*) OR TS=(computational model*) OR TS=(fluid dynamic*) OR TS=(CFD) OR TS=(fluid model*) OR TS=(two phase model*) OR TS=(multi-phase model*) OR TS=(thermodynamic model*) OR TS=(thermal model*) OR TS=(temperature model*) OR TS=(multiphysics model*) OR TS=(computational fluid dynamics)) OR ((TS=(diabetic foot) OR TS=(skin wound)) AND TS=(agent based model*)))*

*NOT*

*(TS = (insole*) OR TS = (shoe*) OR TS = (footwear*) OR TS = (device*) OR TS = (sensor*) OR TS = (deformity) OR TS = (hammer toe*) OR TS = (flatfoot) OR TS = (ostrich foot) OR TS = (amputation) OR TS = (machine learning) OR TS = (deep learning)).*

*2.*	***Scopus:*** *((TITLE-ABS-KEY("plantar" OR "plantar foot" OR "foot sole" OR "plantar soft tissue*" OR "plantar skin" OR "foot skin" OR "diabetic foot" OR "diabetic feet" OR "neuropathic foot" OR "diabetic foot ulcer*" OR "foot ulcer*" OR "diabetic soft tissue*" OR "plantar tissue mechanic*" OR "foot tissue mechanic*))*

*AND*

*(TITLE-ABS-KEY("finite element*" OR "finite element model*" OR "FE model*" OR "FE analysis*" OR "FEA" OR "FEM" OR "mechanobiological model*" OR "constitutive model*" OR "computational model*" OR "fluid dynamic*" OR “CFD” OR "fluid model*" OR "two phase model*" OR "multi phase model*" OR "thermodynamic model*" OR "thermal model*" OR "multiphysics model*" OR "blood flow model*" OR "computational fluid dynamics"))) OR ( TITLE-ABS-KEY ( "diabetic foot" OR "skin wound*" ) AND ( TITLE-ABS-KEY ( "agent based model*" )))*

*AND NOT*

*( TITLE-ABS-KEY ( insole* ) OR TITLE-ABS-KEY ( shoe* ) OR TITLE-ABS-KEY ( footwear* ) OR TITLE-ABS-KEY ( device* ) OR TITLE-ABS-KEY ( sensor* ) OR TITLE-ABS-KEY ( deformity ) OR TITLE-ABS-KEY ( hammer AND toe* ) OR TITLE-ABS-KEY ( flatfoot ) OR TITLE-ABS-KEY ( ostrich AND foot ) OR TITLE-ABS-KEY ( amputation ) OR TITLE-ABS-KEY ( deep learning ) OR TITLE-ABS-KEY ( machine learning ))*

*3.*	***PubMed:*** *(("foot"[MeSH Terms] OR "foot"[All Fields]) AND ("diabetes mellitus"[MeSH Terms] OR ("diabetes"[All Fields] AND "mellitus"[All Fields]) OR "diabetes mellitus"[All Fields] OR "diabetes"[All Fields] OR "diabetic"[All Fields])*

*AND*

*((“finite”[All Fields] AND ("elements"[MeSH Terms] OR "elements"[All Fields] OR "element"[All Fields])) OR ((model[All Fields] OR modeling[All Fields]) AND ("mechanobiological"[All Fields] OR "constitutive"[All Fields] OR "fluid"[All Fields] OR "computational"[All Fields] OR "two phases" OR "three phases"[All Fields] OR "thermodynamic"[All Fields] OR "thermal"[All Fields] OR "multiphysics" OR "blood flow"[All Fields] OR "temperature"[All Fields])))) OR (("diabetic foot"[All Fields] OR "skin wound"[All Fields]) AND ("agent based"[All Fields]))*

*NOT*

*("insole"[All Fields] OR "shoe"[All Fields] OR "footwear"[All Fields] OR "device"[All Fields] OR "sensor"[All Fields] OR "deformity"[All Fields] OR "hammer toe"[All Fields] OR "flatfoot"[All Fields] OR "ostrich foot"[All Fields] OR "amputation"[All Fields] OR "deep learning"[All Fields] OR "machine learning"[All Fields]).”*

- ***Study selection and data extraction process & Screening procedure and eligibility criteria:*** *We have specified the number of reviewers involved at each stage, clearly describing their roles. We now indicate that study selection and data extraction were conducted independently by two reviewers, with discrepancies resolved through discussion (and consultation with a third reviewer where necessary).*

*The study selection process is now described in a stepwise manner, including title/abstract screening followed by full-text assessment, along with the eligibility criteria applied at each stage.*

*Please see Section “Results” – Page 12 for the* ***Study selection and data extraction process and Screening procedure and eligibility criteria*** *revision, please see the text below:*

*“A total of 1631 articles were identified from the three databases (Web of Science: 1173, Scopus: 245, PubMed: 213), and after screening out 324 duplicates and 59 results that were not accessible online, the titles and abstracts of the remaining 1248 articles were reviewed to exclude 1187 articles according to the inclusion and exclusion criteria.*

*A total of 62 articles were retrieved for full-text review. These articles were independently assessed by two reviewers (Li and Tang). In cases where disagreements arose regarding study inclusion, the disputed articles were further evaluated by a third reviewer (Lin) to reach a consensus. Following this literature selection and review process, 33 were excluded for the following reasons: (a) the model was not developed using appropriate geometry medical imaging or experimental data; (b) the study outcomes were not relevant to advancing the understanding of diabetic foot ulcers; and (c) the model lacked adequate validation. In addition, two relevant articles were identified through screening the reference lists of the included studies. Ultimately, a total of 31 articles were included in the final analysis. The overall selection process and results are presented in Fig 1.”*

- ***Data synthesis:*** *We have added a description of how the findings are organised and analyzed in Methods section (e.g., by thematic areas, study types, and model categories).*

*Please see Section “Methods” – Page 10-11 for the* ***Data synthesis*** *revision****:***

*“After the study selection process was completed, the included articles were analysed using a structured grouping approach. Specifically, studies were categorised according to the DFU-related physiological or biomechanical parameters they addressed, as well as the type of computational model employed. Within each group, a comparative analysis was conducted focusing on modelling strategies, material properties, boundary condition settings, as well as the strengths, limitations, and current state of development of the models.”*

- ***Flow Diagram:*** *As suggested, all study selection flow information has been moved to the Results section, see figure 1 in manuscript.*

*Please see Section “Results – Figure 1” for the* ***Flow Diagram*** *revision, please see the text below:*

- ***Quality appraisal:*** *We clarify that no formal quality or risk-of-bias assessment was conducted, consistent with scoping review methodology, and this has been acknowledged as a limitation in the Strengths and Limitations section.*

*Please see Section “Discussion – Strengths and Limitations” – Page 46 for the* ***Quality appraisal*** *revision, please see the text below:*

*“This was scoping review and the findings have the limitation that no formal quality appraisal of the included studies was performed during the review process.”*

**Comment 3:** Results: The study selection flow diagram should be presented in this section.

I believe there is considerable room for improvement in Table 1. At present, key information is missing and there is no data on the country in which each study was conducted, the study design, or the specific objectives of the included studies. The classification system used for the different computational models is not clearly explained in the Methods, and there is no data on the experiments themselves. Likewise, there is no information on the experimental subjects (number and main characteristics), nor on the technical systems used (hardware, software, simulation environment, etc.). Providing these details would greatly enhance the interpretability and usefulness of the results.

**Response 3:** *Thank you very much for your valuable and constructive comments. We have carefully revised the manuscript accordingly.*

1. *Study selection flow diagram: We have now moved the study selection flow diagram to the Results section, as suggested, to improve the clarity and logical structure of the manuscript, see figure 1 in manuscript.*
2. *Revisions to Table 1: We agree that Table 1 required further detail to enhance its interpretability and completeness. Accordingly, we have made the following improvements:*

- *Added a column specifying the study type (e.g., experimental or computational).*
- *Included the country in which each study was conducted.*
- *Introduced a new column outlining each study’s primary focus and objectives.*
- *Added a dedicated “Experimental Setting” column, which provides detailed information on the experimental subjects (including number and key characteristics), as well as the technical systems used (e.g., hardware, software, and simulation environments) and relevant experimental conditions.*

*These revisions aim to provide a more comprehensive overview of the included studies and to improve the overall clarity and usefulness of the results.*

*Thank you again for your helpful suggestions.*

*Please see Section “Results – Fig 1”, Page 13 for the literature selection flow diagram.*

*Please see Section “Results – Table 1” – Page 15- Page 23 for the revised table, see the example rows below:*

**Comment 4:** Discussion: The Discussion does not describe the strengths of this scoping review, and, more importantly, it does not address its limitations. A clear and explicit subsection on strengths and limitations is essential in any review and should be incorporated, reflecting, for example, potential biases in the search and selection process, the lack of quality appraisal (if applicable), and any restrictions in scope (languages, time period, types of studies, or models included).

**Response 4:** *Thank you very much for your insightful and constructive suggestion. We fully agree that a clear discussion of both strengths and limitations is essential for improving the transparency and rigor of the review.*

*In response, we have added a dedicated subsection entitled* ***“Strengths and Limitations”*** *in the* Discussion *section. This new subsection explicitly outlines the main strengths of our scoping review, as well as its limitations, including potential biases in the search and selection process, the absence of formal quality appraisal, and any restrictions related to scope (e.g., language, time frame, and types of included studies).*

*Please see Section “Discussions – Strengths and Limitations” – Page 46:*

*“This study has several important strengths. First, this is the first scoping review to systematically examine a wide range of foot models, encompassing different modelling strategies and model types. Based on a comprehensive review of existing literature, this review not only summarizes the current state of development of various foot models but also provides an in-depth analysis of their modelling approaches, application contexts, and remaining limitations. It highlights the potential of computational modelling techniques to advance understanding the mechanisms of DFU, provides a clear overall framework for research progress in this field, and helps identify potential directions for future research.*

*However, this review still has some limitations. First, the literature search was conducted up to March 2025, and therefore studies published after were not included. Second, the search was restricted to three literature databases and to accessible publications written in English, introducing the possibility of language and selection bias. In addition, this review focused primarily on studies involving computational modelling and did not include purely clinical trials or experimenyal studies. Finally, no formal quality appraisal of the included studies was performed during the review process.”*
