# Supplementary material for: A scoping review of computational models of the diabetic foot
Source: PLoS One. 2026 Jun 15;21(6):e0351638. doi: 10.1371/journal.pone.0351638 (PMC13268164; doi:10.1371/journal.pone.0351638)
Supplement: Peer review history

# Peer review: Decision Letter 0

**DOI:** 10.1371/journal.pone.0351638.r001

**Authors:** Fei Yan

23 Dec 2025

PONE-D-25-63063

A scoping review of computational models of the diabetic foot

PLOS One

Dear Dr. Li,

Thank you for submitting your manuscript to PLOS ONE. After careful consideration, we feel that it has merit but does not fully meet PLOS ONE’s publication criteria as it currently stands. Therefore, we invite you to submit a revised version of the manuscript that addresses the points raised during the review process.

Please submit your revised manuscript by Feb 06 2026 11:59PM. If you will need more time than this to complete your revisions, please reply to this message or contact the journal office at plosone@plos.org. When you're ready to submit your revision, log on to https://www.editorialmanager.com/pone/ and select the 'Submissions Needing Revision' folder to locate your manuscript file.

Please include the following items when submitting your revised manuscript:

- A letter that responds to each point raised by the academic editor and reviewer(s). You should upload this letter as a separate file labeled 'Response to Reviewers'.
- A marked-up copy of your manuscript that highlights changes made to the original version. You should upload this as a separate file labeled 'Revised Manuscript with Track Changes'.
- An unmarked version of your revised paper without tracked changes. You should upload this as a separate file labeled 'Manuscript'.

If you would like to make changes to your financial disclosure, please include your updated statement in your cover letter. Guidelines for resubmitting your figure files are available below the reviewer comments at the end of this letter.

If applicable, we recommend that you deposit your laboratory protocols in protocols.io to enhance the reproducibility of your results. Protocols.io assigns your protocol its own identifier (DOI) so that it can be cited independently in the future. For instructions see: https://journals.plos.org/plosone/s/submission-guidelines#loc-laboratory-protocols. Additionally, PLOS ONE offers an option for publishing peer-reviewed Lab Protocol articles, which describe protocols hosted on protocols.io. Read more information on sharing protocols at https://plos.org/protocols?utm_medium=editorial-email&utm_source=authorletters&utm_campaign=protocols.

We look forward to receiving your revised manuscript.

Kind regards,

Fei Yan

Academic Editor

PLOS One

Journal Requirements:

When submitting your revision, we need you to address these additional requirements.

1. Please ensure that your manuscript meets PLOS ONE's style requirements, including those for file naming. The PLOS ONE style templates can be found at

https://journals.plos.org/plosone/s/file?id=wjVg/PLOSOne_formatting_sample_main_body.pdf and

https://journals.plos.org/plosone/s/file?id=ba62/PLOSOne_formatting_sample_title_authors_affiliations.pdf

2. Please update your submission to use the PLOS LaTeX template. The template and more information on our requirements for LaTeX submissions can be found at http://journals.plos.org/plosone/s/latex.

3. Please upload a new copy of Figure 3 as the detail is not clear. Please follow the link for more information:  https://journals.plos.org/plosone/s/figures

4. Please remove your figures from within your manuscript file, leaving only the individual TIFF/EPS image files, uploaded separately. These will be automatically included in the reviewers’ PDF.

5. If the reviewer comments include a recommendation to cite specific previously published works, please review and evaluate these publications to determine whether they are relevant and should be cited. There is no requirement to cite these works unless the editor has indicated otherwise.

[Note: HTML markup is below. Please do not edit.]

Reviewers' comments:

Reviewer's Responses to Questions

**Comments to the Author**

1. Is the manuscript technically sound, and do the data support the conclusions?

The manuscript must describe a technically sound piece of scientific research with data that supports the conclusions. Experiments must have been conducted rigorously, with appropriate controls, replication, and sample sizes. The conclusions must be drawn appropriately based on the data presented.

Reviewer #1: Yes

Reviewer #2: Partly

**********

2. Has the statistical analysis been performed appropriately and rigorously?

Reviewer #1: Yes

Reviewer #2: N/A

**********

3. Have the authors made all data underlying the findings in their manuscript fully available?

The PLOS Data policy requires authors to make all data underlying the findings described in their manuscript fully available without restriction, with rare exception (please refer to the Data Availability Statement in the manuscript PDF file). The data should be provided as part of the manuscript or its supporting information, or deposited to a public repository. For example, in addition to summary statistics, the data points behind means, medians and variance measures should be available. If there are restrictions on publicly sharing data—e.g. participant privacy or use of data from a third party—those must be specified.

Reviewer #1: Yes

Reviewer #2: No

**********

4. Is the manuscript presented in an intelligible fashion and written in standard English?

PLOS ONE does not copyedit accepted manuscripts, so the language in submitted articles must be clear, correct, and unambiguous. Any typographical or grammatical errors should be corrected at revision, so please note any specific errors here.

Reviewer #1: Yes

Reviewer #2: Yes

**********

5. Review Comments to the Author

Please use the space provided to explain your answers to the questions above. You may also include additional comments for the author, including concerns about dual publication, research ethics, or publication ethics. (Please upload your review as an attachment if it exceeds 20,000 characters)

Reviewer #1: The manuscript presents a well-structured and timely scoping review on computational modeling of diabetic foot ulcers (DFU), a topic of high clinical and engineering relevance. The review identifies and categorizes five model types (mechanical, thermal, vascular/nerve, multiphysics, cellular), which provides a useful framework for readers. The inclusion of a PRISMA flow diagram and detailed tables enhances transparency and reproducibility. Some issues needs to be addressed.

1. Scope and Search Strategy: The justification for the exclusion criteria, particularly the broad exclusion of studies from fields like "medicine, pharmacology, veterinary, chemistry…" requires clarification. While the intent to focus on engineering and computational aspects is clear, this blanket exclusion might omit some biomechanical studies published in medical journals. Please refine or better justify these criteria to ensure key interdisciplinary work is captured. Additionally, the search strategy is comprehensive, but including terms like "diabetic foot syndrome," "plantar pressure," or "tissue mechanics" could further optimize recall.

2. Please provide further explanation to clarify the distinction between "vascular and nerve system models" and "multiphysics models." For instance, some models categorized as "multiphysics" (e.g., Mithraratne et al., 2012) also involve substantial vascular/blood flow analysis.

3.To enhance the utility of the summary table (Table 1), it is recommended to add a column for "Primary Research Theme/Objective" for each study.

Furthermore, a brief indication of the model's validation status (e.g., "experimentally validated," "compared with literature," "not validated") in the same table or in the analysis would provide valuable insight into the methodological rigor of the field.

4. Please expand the Discussion section to specifically address the implications of these limitations for clinical prediction and prevention. For example, how might the inability to model deep tissue stress concentrations or failure hinder the development of personalized ulcer risk assessment tools? What would be the potential clinical value of a validated, multi-layer model with accurate failure mechanics?

5. P2, Abstract: "couple these together to to develop" – remove duplicate "to"

6. P5: "objectivities" should likely be "objectives"

Reviewer #2: Dear authors,

Thank you for the opportunity to review your manuscript. Diabetic foot is one of my main areas of research, although my expertise focuses specifically on nursing care and clinical management of the diabetic foot, rather than on the technical and computational aspects that are the core topic of this work. Therefore, the comments I will provide are mainly related to the methodology used in your scoping review rather than to the specific computational results, which fall outside my expertise.

1-Introduction

I consider the Introduction to be appropriate in terms of contextualising diabetic foot as a pathology in the international setting and highlighting its clinical relevance. However, it is not sufficiently informative regarding the main types of computational models that have been used to study this condition, nor the existing discrepancies or limitations of these models to date. As I understand it, clarifying these issues is precisely one of the aims of your review.

For example, you later use a classification of models based on “different focused factors” or according to whether they range from “macroscopic to microscopic”. These criteria should be introduced and briefly explained in the Introduction (or at least clearly defined before the Results), so that readers can fully understand and correctly interpret the subsequent classification and findings.

2. Methods

The authors should review and implement the PRISMA-ScR recommendations for scoping reviews. I highlight below several aspects of the methods section that need to be improved or clarified:

The guiding research question that underpins the review should be stated explicitly, and, if possible, the framework used to formulate it should be indicated (e.g. PICO, PEO, PECO, etc.).

It should be clearly specified which search terms were MeSH (or equivalent controlled vocabulary) and which were free-text terms. The full search strategies (search equations) for each database, including the exact combinations of terms and Boolean operators, as well as the date limits and final search dates for each database, should be reported.

The number of reviewers involved in each stage of the process should be specified, together with their roles. It should be made clear whether study selection and data extraction were performed in pairs (independently and in duplicate) and how discrepancies were resolved (e.g. consensus, third reviewer). Please also indicate whether any software or assisted systems were used to support these processes. The snowballing procedure that you mention should likewise be described in greater detail (how it was conducted, which references were followed, selection rules, etc.). All flow information (study selection process) should be reported in the Results section, not in the Methods.

At no point is the study selection process clearly explained. It should be specified whether records were first screened by title and abstract and then assessed in full text, and according to which criteria. It is also not clear which variables were extracted from each included study, whether a pre-defined data extraction form was used, and how the data extraction by pairs (if applicable) was organised.

As this is a scoping review, it is acceptable not to perform a formal quality or risk-of-bias assessment of the included studies, although this is obviously a limitation that should be acknowledged. In any case, the Methods should explain how the expected findings will be organised and synthesised (e.g. thematic areas, types of studies to be included, types of models, outcomes, etc.).

In summary, as mentioned earlier, I strongly recommend consulting and adhering to PRISMA-ScR guidelines for this type of review and ensuring that all core methodological elements are reported accordingly.

3. Results

The study selection flow diagram should be presented in this section.

I believe there is considerable room for improvement in Table 1. At present, key information is missing: there are no data on the country in which each study was conducted, the study design, or the specific objectives of the included studies. The classification system used for the different computational models is not clearly explained in the Methods, and there are no data on the experiments themselves. Likewise, there is no information on the experimental subjects (number and main characteristics), nor on the technical systems used (hardware, software, simulation environment, etc.). Providing these details would greatly enhance the interpretability and usefulness of the results.

4. Discussion

The Discussion does not describe the strengths of this scoping review, and, more importantly, it does not address its limitations. A clear and explicit subsection on strengths and limitations is essential in any review and should be incorporated, reflecting, for example, potential biases in the search and selection process, the lack of quality appraisal (if applicable), and any restrictions in scope (languages, time period, types of studies, or models included).

I trust that these comments will be taken into account as opportunities for improvement. Best regards

**********

6. PLOS authors have the option to publish the peer review history of their article (what does this mean?). If published, this will include your full peer review and any attached files.

If you choose “no”, your identity will remain anonymous but your review may still be made public.

**Do you want your identity to be public for this peer review?**    For information about this choice, including consent withdrawal, please see our Privacy Policy.

Reviewer #1: No

Reviewer #2: **Yes:**   Héctor González-de la Torre

**********

[NOTE: If reviewer comments were submitted as an attachment file, they will be attached to this email and accessible via the submission site. Please log into your account, locate the manuscript record, and check for the action link "View Attachments". If this link does not appear, there are no attachment files.]

To ensure your figures meet our technical requirements, please review our figure guidelines: https://journals.plos.org/plosone/s/figures

You may also use PLOS’s free figure tool, NAAS, to help you prepare publication quality figures: https://journals.plos.org/plosone/s/figures#loc-tools-for-figure-preparation.

NAAS will assess whether your figures meet our technical requirements by comparing each figure against our figure specifications.

# Author comment: Author response to Decision Letter 1

**DOI:** 10.1371/journal.pone.0351638.r002

20 May 2026

Please see all the responses in the file "Response to Reviewers", thank you.

Attachment Submitted filename: Response to Reviewers.docx

# Peer review: Decision Letter 1

**DOI:** 10.1371/journal.pone.0351638.r003

**Authors:** Fei Yan

1 Jun 2026

A scoping review of computational models of the diabetic foot

PONE-D-25-63063R1

Dear Dr. Li,

We’re pleased to inform you that your manuscript has been judged scientifically suitable for publication and will be formally accepted for publication once it meets all outstanding technical requirements.

Within one week, you’ll receive an e-mail detailing the required amendments. When these have been addressed, you’ll receive a formal acceptance letter and your manuscript will be scheduled for publication.

An invoice will be generated when your article is formally accepted. Please note, if your institution has a publishing partnership with PLOS and your article meets the relevant criteria, all or part of your publication costs will be covered. Please make sure your user information is up-to-date by logging into Editorial Manager at Editorial Manager® and clicking the ‘Update My Information' link at the top of the page. For questions related to billing, please contact billing support.

If your institution or institutions have a press office, please notify them about your upcoming paper to help maximize its impact. If they’ll be preparing press materials, please inform our press team as soon as possible -- no later than 48 hours after receiving the formal acceptance. Your manuscript will remain under strict press embargo until 2 pm Eastern Time on the date of publication. For more information, please contact onepress@plos.org.

Kind regards,

Fei Yan

Academic Editor

PLOS One

Additional Editor Comments (optional):

Reviewers' comments:

Reviewer's Responses to Questions

**Comments to the Author**

1. If the authors have adequately addressed your comments raised in a previous round of review and you feel that this manuscript is now acceptable for publication, you may indicate that here to bypass the “Comments to the Author” section, enter your conflict of interest statement in the “Confidential to Editor” section, and submit your "Accept" recommendation.

Reviewer #1: All comments have been addressed

Reviewer #2: All comments have been addressed

**********

2. Is the manuscript technically sound, and do the data support the conclusions?

The manuscript must describe a technically sound piece of scientific research with data that supports the conclusions. Experiments must have been conducted rigorously, with appropriate controls, replication, and sample sizes. The conclusions must be drawn appropriately based on the data presented.

Reviewer #1: Yes

Reviewer #2: Yes

**********

3. Has the statistical analysis been performed appropriately and rigorously?

Reviewer #1: Yes

Reviewer #2: N/A

**********

4. Have the authors made all data underlying the findings in their manuscript fully available?

The PLOS Data policy requires authors to make all data underlying the findings described in their manuscript fully available without restriction, with rare exception (please refer to the Data Availability Statement in the manuscript PDF file). The data should be provided as part of the manuscript or its supporting information, or deposited to a public repository. For example, in addition to summary statistics, the data points behind means, medians and variance measures should be available. If there are restrictions on publicly sharing data—e.g. participant privacy or use of data from a third party—those must be specified.

Reviewer #1: Yes

Reviewer #2: Yes

**********

5. Is the manuscript presented in an intelligible fashion and written in standard English?

PLOS ONE does not copyedit accepted manuscripts, so the language in submitted articles must be clear, correct, and unambiguous. Any typographical or grammatical errors should be corrected at revision, so please note any specific errors here.

Reviewer #1: Yes

Reviewer #2: Yes

**********

6. Review Comments to the Author

Please use the space provided to explain your answers to the questions above. You may also include additional comments for the author, including concerns about dual publication, research ethics, or publication ethics. (Please upload your review as an attachment if it exceeds 20,000 characters)

Reviewer #1: The authors have provided detailed responses to all the questions and concerns raised by the reviewers.

Reviewer #2: Dear Authors,

Thank you for allowing me to review your manuscript again. I believe you have addressed the reviewers’ comments, and the manuscript has improved in both transparency and methodological rigor. From my perspective, it is now suitable for publication.

Best regards,

**********

7. PLOS authors have the option to publish the peer review history of their article (what does this mean?). If published, this will include your full peer review and any attached files.

If you choose “no”, your identity will remain anonymous but your review may still be made public.

**Do you want your identity to be public for this peer review?**    For information about this choice, including consent withdrawal, please see our Privacy Policy.

Reviewer #1: No

Reviewer #2: **Yes:**   Héctor González-de la Torre

**********

# Editor report: Acceptance letter

**DOI:** 10.1371/journal.pone.0351638.r004

**Authors:** Fei Yan

PONE-D-25-63063R1

PLOS One

Dear Dr. Li,

I'm pleased to inform you that your manuscript has been deemed suitable for publication in PLOS One. Congratulations! Your manuscript is now being handed over to our production team.

At this stage, our production department will prepare your paper for publication. This includes ensuring the following:

* All references, tables, and figures are properly cited

* All relevant supporting information is included in the manuscript submission,

* There are no issues that prevent the paper from being properly typeset

You will receive further instructions from the production team, including instructions on how to review your proof when it is ready. Please keep in mind that we are working through a large volume of accepted articles, so please give us a few days to review your paper and let you know the next and final steps.

Lastly, if your institution or institutions have a press office, please let them know about your upcoming paper now to help maximize its impact. If they'll be preparing press materials, please inform our press team within the next 48 hours. Your manuscript will remain under strict press embargo until 2 pm Eastern Time on the date of publication. For more information, please contact onepress@plos.org.

You will receive an invoice from PLOS for your publication fee after your manuscript has reached the completed accept phase. If you receive an email requesting payment before acceptance or for any other service, this may be a phishing scheme. Learn how to identify phishing emails and protect your accounts at https://explore.plos.org/phishing.

If we can help with anything else, please email us at customercare@plos.org.

Thank you for submitting your work to PLOS ONE and supporting open access.

Kind regards,

PLOS ONE Editorial Office Staff

on behalf of

Dr. Fei Yan

Academic Editor

PLOS One
